# Predicting Antidepressant Treatment Response From Cortical Structure on MRI: A Mega‐Analysis From the ENIGMA‐MDD Working Group

**DOI:** 10.1002/hbm.70053

**Published:** 2025-01-06

**Authors:** Maarten G. Poirot, Daphne E. Boucherie, Matthan W. A. Caan, Roberto Goya‐Maldonado, Vladimir Belov, Emmanuelle Corruble, Romain Colle, Baptiste Couvy‐Duchesne, Toshiharu Kamishikiryo, Hotaka Shinzato, Naho Ichikawa, Go Okada, Yasumasa Okamoto, Ben J. Harrison, Christopher G. Davey, Alec J. Jamieson, Kathryn R. Cullen, Zeynep Başgöze, Bonnie Klimes‐Dougan, Bryon A. Mueller, Francesco Benedetti, Sara Poletti, Elisa M. T. Melloni, Christopher R. K. Ching, Ling‐Li Zeng, Joaquim Radua, Laura K. M. Han, Neda Jahanshad, Sophia I. Thomopoulos, Elena Pozzi, Dick J. Veltman, Lianne Schmaal, Paul M. Thompson, Henricus G. Ruhe, Liesbeth Reneman, Anouk Schrantee

**Affiliations:** ^1^ Amsterdam UMC, Department of Radiology and Nuclear Medicine University of Amsterdam Amsterdam the Netherlands; ^2^ Department of Biomedical Engineering and Physics Amsterdam UMC,University of Amsterdam Amsterdam the Netherlands; ^3^ Amsterdam Neuroscience, Brain Imaging Amsterdam the Netherlands; ^4^ Division of Radiology and Nuclear Medicine, Computational Radiology and Artificial Intelligence (CRAI) Oslo University Hospital Oslo Norway; ^5^ Laboratory of Systems Neuroscience and Imaging in Psychiatry (SNIP‐Lab), Department of Psychiatry and Psychotherapy University Medical Center Göttingen (UMG) Göttingen Germany; ^6^ MOODS Team, INSERM 1018, Centre de Recherche en Epidémiologie et Santé Des Populations Université Paris‐Saclay, Faculté de Médecine Paris‐Saclay, Le Kremlin Bicêtre Le Kremlin‐Bicêtre France; ^7^ Service Hospitalo‐Universitaire de Psychiatrie de Bicêtre, Mood Center Paris Saclay, Assistance Publique‐Hôpitaux de Paris Hôpitaux Universitaires Paris‐Saclay, Hôpital de Bicêtre, Le Kremlin Bicêtre Le Kremlin‐Bicêtre France; ^8^ Paris‐Saclay University Le Kremlin‐Bicêtre France; ^9^ Institute for Molecular Bioscience the University of Queensland St Lucia Queensland Australia; ^10^ Sorbonne University Paris Brain Institute—ICM, CNRS, Inria, Inserm, AP‐HP, Hôpital de la Pitié Salpêtrière Paris France; ^11^ Department of Psychiatry and Neurosciences. Graduate School of Biomedical & Health Sciences Hiroshima University Hiroshima Japan; ^12^ Department of Neuropsychiatry, Graduate School of Medicine University of the Ryukyus Okinawa Japan; ^13^ Deloitte Analytics R&D, Deloitte Touche Tohmatsu LLC Tokyo Japan; ^14^ Department of Psychiatry The University of Melbourne Melbourne Australia; ^15^ University of Minnesota Minneapolis Minnesota USA; ^16^ Division of Neuroscience, Psychiatry & Clinical Psychobiology Unit IRCCS San Raffaele Scientific Institute Milano Italy; ^17^ Vita‐Salute San Raffaele University Milano Italy; ^18^ Imaging Genetics Center, Mark & Mary Stevens Neuroimaging and Informatics Institute, Keck School of Medicine University of Southern California Los Angeles California USA; ^19^ College of Intelligence Science and Technology National University of Defense Technology Changsha China; ^20^ IDIBAPS, CIBERSAM Instituto de Salud Carlos III Barcelona Spain; ^21^ Centre for Youth Mental Health The University of Melbourne Parkville Victoria Australia; ^22^ Orygen Parkville Victoria Australia; ^23^ Department of Psychiatry Amsterdam UMC, Location VUmc Amsterdam the Netherlands; ^24^ Department of Psychiatry Nijmegen the Netherlands; ^25^ Donders Institute for Brain, Cognition and Behavior Radboud University Nijmegen the Netherlands

**Keywords:** antidepressant treatment response, ENIGMA, machine learning, magnetic resonance imaging, major depressive disorder, mega‐analysis, Radiomics

## Abstract

Accurately predicting individual antidepressant treatment response could expedite the lengthy trial‐and‐error process of finding an effective treatment for major depressive disorder (MDD). We tested and compared machine learning‐based methods that predict individual‐level pharmacotherapeutic treatment response using cortical morphometry from multisite longitudinal cohorts. We conducted an international analysis of pooled data from six sites of the ENIGMA‐MDD consortium (*n* = 262 MDD patients; age = 36.5 ± 15.3 years; 154 (59%) female; mean response rate = 57%). Treatment response was defined as a ≥ 50% reduction in symptom severity score after 4–12 weeks post‐initiation of antidepressant treatment. Structural MRI was acquired before, or < 14 days after, treatment initiation. The cortex was parcellated using FreeSurfer, from which cortical thickness and surface area were measured. We tested several machine learning pipeline configurations, which varied in (i) the way we presented the cortical data (i.e., average values per region of interest, as a vector containing voxel‐wise cortical thickness and surface area measures, and as cortical thickness and surface area projections), (ii) whether we included clinical data, and the (iii) machine learning model (i.e., gradient boosting, support vector machine, and neural network classifiers) and (iv) cross‐validation methods (i.e., k‐fold and leave‐one‐site‐out) we used. First, we tested if the overall predictive performance of the pipelines was better than chance, with a corrected 10‐fold cross‐validation permutation test. Second, we compared if some machine learning pipeline configurations outperformed others. In an exploratory analysis, we repeated our first analysis in three subpopulations, namely patients (i) from a single site, (ii) with comparable response rates, and (iii) showing the least (first quartile) and the most (fourth quartile) treatment response, which we call the extreme (non‐)responders subpopulation. Finally, we explored the effect of including subcortical volumetric data on model performance. Overall, performance predicting antidepressant treatment response was not significantly better than chance (balanced accuracy = 50.5%; *p* = 0.66) and did not vary with alternative pipeline configurations. Exploratory analyses revealed that performance across models was only significantly better than chance in the extreme (non‐)responders subpopulation (balanced accuracy = 63.9%, *p* = 0.001). Including subcortical data did not alter the observed model performance. Cortical structural MRI alone could not reliably predict individual pharmacotherapeutic treatment response in MDD. None of the used machine learning pipeline configurations outperformed the others. In exploratory analyses, we found that predicting response in the extreme (non‐)responders subpopulation was feasible on both cortical data alone and combined with subcortical data, which suggests that specific MDD subpopulations may exhibit response‐related patterns in structural data. Future work may use multimodal data to predict treatment response in MDD.

## Graphical abstract

In this mega‐analysis of cortical structural MRI in 262 individuals with major depression disorder (MDD), pooled from six sites of the ENIGMA‐MDD consortium, we tested various machine‐learning pipeline configurations. We provide compelling evidence that cortical structural MRI alone is not a reliable predictor of individualized pharmacotherapeutic treatment response in MDD.

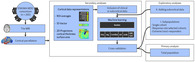

**Summary**

- FreeSurfer‐derived cortical thickness and surface area measures showed no predictive value for pharmacotherapeutic treatment response in major depressive disorder in the current sample of the population at large.
- Classification performance was not dependent on machine learning pipeline configuration, that is, cortical data representation, the inclusion of clinical data, the machine learning method used, or the cross‐validation scheme used.
- Exploratory analyses suggested that response could be predicted from cortical structural data for a specific subpopulation of MDD patients, that is, in the 25% least and most responsive categories.

## 1 Introduction

Major depressive disorder (MDD) is a highly debilitating psychiatric disorder with a high and growing lifetime prevalence of ~20% (Proudman, Greenberg, and Nellesen 2021). MDD is the second leading contributor to disability, with annual worldwide cost estimated at US$ 1 trillion in lost productivity alone (Bromet et al. 2011; Chodavadia et al. 2023; Greenberg et al. 2021). The first line of treatment often consists of antidepressant treatment because of its established efficacy, and known side‐effects and safety profile (Cipriani et al. 2018; Santarsieri and Schwartz 2015). However, individual response to antidepressant treatment is highly variable among patients, and there remains no validated predictor of individual treatment effect. Therefore, antidepressant treatment planning resorts to a trial‐and‐error approach and initial treatment only achieves significant symptom relief in one‐third of patients (Rush et al. 2009). This means that individuals with MDD are frequently subjected to multiple futile treatments, which prolongs disease burden and risks adverse effects such as further worsening of symptoms and risk of suicide (Zisook et al. 2009). To improve treatment planning and reduce disease burden, early predictors of treatment efficacy are needed.

Neuroimaging techniques, such as magnetic resonance imaging (MRI), have substantially improved our understanding of brain alterations in MDD. For example, large‐scale structural MRI analyses have shown that MDD is associated with patterns of thinner cortical gray matter in the orbitofrontal cortex, anterior and posterior cingulate, and insula and temporal lobes, as well as lower cortical surface area in frontal regions and in primary and higher‐order visual, somatosensory and motor areas compared to healthy volunteers (Schmaal et al. 2017). Furthermore, structural MRI can differentiate treatment‐resistant depression from other forms of MDD (Klok et al. 2019). Although multiple reviews also postulate the predictive value of such biomarkers from structural MRI (Fonseka, MacQueen, and Kennedy 2018; Schrantee, Ruhé, and Reneman 2020), the generally low to moderate effect sizes observed in these studies impede the clinical translatability of these predictors. A promising avenue to enable individual‐level inference is the use of radiomics—the extraction of a large number of features from medical images—and machine learning methods. A recent review of studies that applied deep learning methods to a wide range of features (clinical, demographic, genetic, functional neuroimaging) to predict treatment response in MDD found that they outperform regression models, achieving relatively high area‐under‐the‐curve (AUC) (Squarcina et al. 2021). Therefore, MRI predictors, combined with machine learning or deep learning, may support the search for effective treatment for MDD.

Unfortunately, many studies that aimed to predict treatment response in MDD were restricted by small sample sizes or small training or test sets, with a high risk of overestimating the performance of the predictive models (Cohen et al. 2021; Flint et al. 2021; Sajjadian et al. 2021). Moreover, the variation in techniques and analysis approaches is a major challenge in translating potential predictive biomarkers for clinical application (Schrantee, Ruhé, and Reneman 2020). Collaborative efforts, such as the Enhancing Neuroimaging Genetics through Meta‐Analysis (ENIGMA) MDD consortium, offer a vital solution to overcome this challenge. This global initiative has pooled MRI and demographic data from existing samples around the world from 52 independent sites in 17 countries and six continents. By applying standardized processing, quality control, and analysis procedures to these large‐scale data, ENIGMA‐MDD is addressing some of the core challenges facing prior smaller‐scale studies of MDD (Schrantee, Ruhé, and Reneman 2020). In addition, this approach enables the modeling of individual patient‐level predictors rather than site‐averaged data, which is more potent than traditional meta‐analyses (Harrewijn et al. 2021). Such large‐scale approaches with global representation are crucial for identifying reliable and generalizable brain alterations associated with MDD (Shrout and Rodgers 2018), as previous studies from this consortium have demonstrated (Schmaal et al. 2020; Thompson et al. 2020).

**FIGURE 1:**
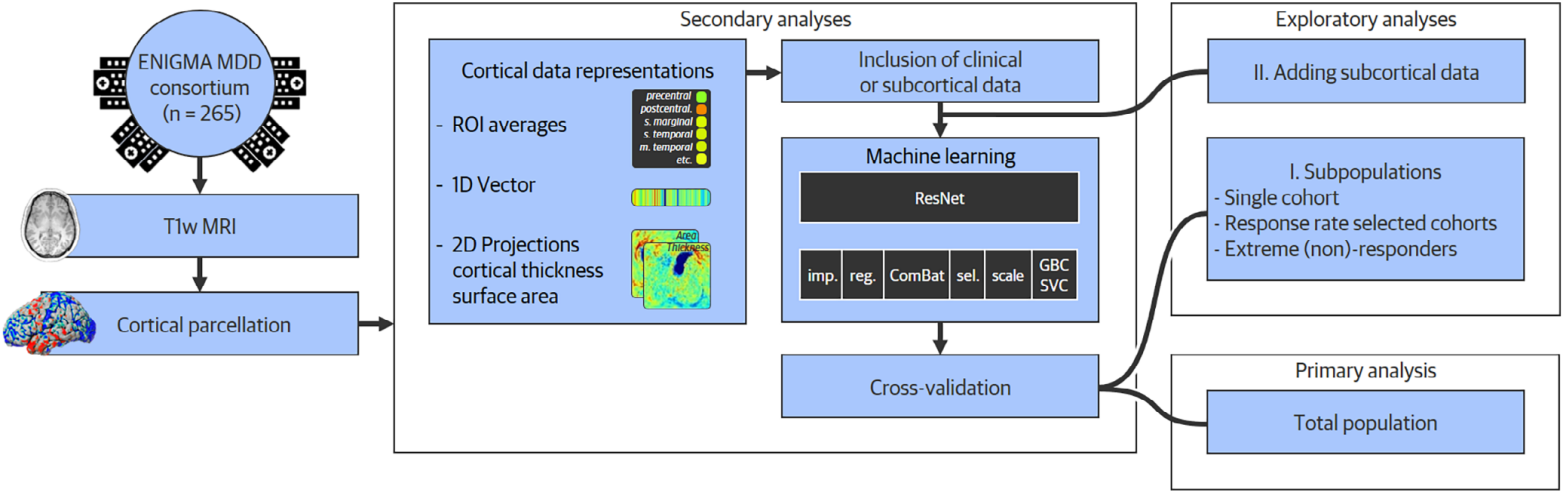
Processing and analysis pipeline. The complete analysis pipeline is presented from left to right, starting with data acquisition and preprocessing steps. Four steps, part of the machine learning pipeline, follow these. We tested various machine learning pipeline configurations for the four steps presented in our secondary analyses. Finally, the model is trained and tested, initially on the full population and subsequently in exploratory analyses on three subpopulations. Finally, we test if adding subcortical data improves predictive performance for our secondary exploratory analysis. ENIGMA MDD, Enhancing Neuroimaging Genetics through Meta‐Analysis Major Depressive Disorder Working Group; GBC, Gradient boosting classifier; imp., imputation; sel, feature selection; SVC, support vector classifier; reg., regressing out confounders; ROI, Region of interest; T1w MRI, T1‐weighted Magnetic Resonance Imaging.

In this study, we tested the hypothesis that machine learning approaches—applied to pre‐treatment cortical structural MRI‐derived measures—can predict pharmacotherapeutic treatment response better than chance. For our secondary analyses, we hypothesized that more advanced predictive modeling pipeline configurations (e.g., a deep learning residual network) would outperform more classical machine learning approaches (e.g., a support vector machine). We compared several machine learning pipeline configurations varying in four aspects: the way we presented the cortical structural data, the inclusion of clinical information, and in the machine learning algorithm and cross‐validation (CV) scheme used (see Figure 1). Together, these modeling variations cover a range of common machine learning approaches discussed in the literature, which allows a thorough investigation of the effect of model configurations on predictive performance. We also conducted exploratory analyses on three subpopulations to further evaluate the modeling configurations. We selected these subpopulations to either increase the homogeneity of the sample (subpopulations I and II) or to improve the homogeneity of the dichotomous outcome labels (subpopulation III). We identified the following subpopulations: (i) participants from a single cohort, (ii) participants from cohorts with comparable mean response rates, and (iii) participants who showed the most and least percentage changes in symptom severity in response to antidepressant treatment.

## 2 Materials and Methods

The analyses described below (with exception of the exploratory analyses) were pre‐registered in an analysis plan before receiving the data. The plan was shared with and approved by the ENIGMA MDD consortium, and is attached to the Supplementary Methods in Data S1. All code related to this work is available through our online repository (Poirot et al. 2023).

### 2.1 Population

Six international ENIGMA MDD Working Group cohorts contributed data to our analysis (Table 1). All participating sites obtained approval from their institutional review boards and ethics committees and acquired written informed consent from all participants.

**TABLE 1:** Demographic, clinical and outcome characteristics across cohorts.

Characteristic	Total		Cohorts												Subpopulations^a^			
	Total (n = 262)		AFFDIS (*n* = 16)		DEP‐ARREST‐CLIN (*n* = 57)		Hiroshima cohort (*n* = 92)		Melbourne (*n* = 49)		Minnesota (*n* = 13)		Milano OSR (*n* = 35)		Response Rate Selected (*n* = 157)		Extreme (non‐) responders (*n* = 132)	
	Mean	SD	Mean	SD	Mean	SD	Mean	SD	Mean	SD	Mean	SD	Mean	SD	Mean	SD	Mean	SD
Age (years)	36.5	15.3	44.1	14.1	34.1	12.7	43.0	11.6	19.6	3.0	15.0	2.2	51.3	9.6	35.8	14.9	34.3	14.8
Treatment duration (weeks)	8.3	3.2	5.1	0.7	12.0	0.0	6.0	0.0	12.0	0.0	9.9	1.9	4.3	0.7	7.8	2.9	8.9	3.3
Normalized pre‐treatment symptom severity	0.04	0.99	−0.85	1.20	0.79	0.78	−0.31	0.79	0.28	0.72	0.15	0.95	−0.25	1.12	−0.18	0.89	0.03	0.98
Age at first depressive episode (years)	30.3	14.8	0.4	15.7	0.3	11.7	0.4	0.1	15.7	2.7	11.2	2.6	36.5	11.1	31.4	15.7	27.2	13.8

	*N*	%	*N*	%	*N*	%	*N*	%	*N*	%	*N*	%	*N*	%	*N*	%	*N*	%
Female	154	59	5	31	39	68	47	51	33	67	9	69	21	60	85	54	81	61
MDD is recurrent	160	61	15	94	29	51	46	50	31	63	9	69	30	86	92	59	83	63
Responds to treatment	149	57	6	38	48	84	45	49	22	45	8	62	20	57	73	47	66	50
Uses SSRI	194	74	9	56	0	0	91	99	49	100	13	100	32	91	149	95	91	69
Uses SNRI	68	26	3	19	57	100	0	0	0	0	0	0	8	23	3	2	42	32
Uses atypical antidepressant	16	6	8	50	0	0	1	1	0	0	0	0	7	20	9	6	5	4
Scanned after treatment initiation	137	52	13	81	0	0	86	93	0	0	0	0	27	100	99	63	57	43
In the response rate selected subpopulation	157	60	16	100	0	0	92	100	49	100	0	0	0	0	157	100	67	51
In the extreme (non‐)responders subpopulation	132	50	8	50	36	63	33	36	26	53	11	85	18	51	67	43	132	100
Symptom severity scoring method			MADRS		HDRS		HDRS		MADRS		BDI		HDRS		—		—	

*Note:* Characteristics are shown for the total population, each of the included cohorts, and for the subpopulations used in our exploratory analyses. In the first part of the table, the mean and SD per characteristic are provided; in the second part of the table, the participant numbers and the percentage per characteristic are provided.

Abbreviations: BDI, Beck Depression Inventory; HDRS, Hamilton Depression Rating Scale; MADRS, Montgomery–Åsberg Depression Rating Scale; SNRI, selective serotonin and norepinephrine reuptake inhibitor; MDD, major depressive disorder; SSRI, selective serotonin reuptake inhibitor.

^a^ The third subpopulation analyzed, the single cohort subpopulation, was omitted from this table as it consisted of patients from the Hiroshima cohort only.

Inclusion criteria were patients with MDD, for whom pharmacological treatment with any antidepressant was deemed necessary, the presence of both pre‐treatment and follow‐up measurement of symptom severity, and a pre‐treatment structural MRI scan. If patients switched medication, a treatment duration of ≥ 4 weeks with the new medication before the follow‐up measurement was required. Patients were not required to be free of antidepressant medication at the time of scanning. Exclusion criteria consisted of the use of tricyclic antidepressants or quetiapine, a treatment duration of < 4 weeks, and a pre‐treatment structural MRI scan obtained > 14 days after antidepressant treatment initiation. To assess the influence of the treatment duration threshold on our findings, we additionally conducted a sensitivity analysis where we restricted our primary analyses to patients with a treatment duration of ≥ 8 weeks, the results of which can be found in Table S1. In addition, to assess the influence of the used baseline MRI cut‐off values of 14 days, we additionally conducted a sensitivity analysis where we repeated our primary analyses but excluded all participants with a baseline MRI scan obtained > 7 days after treatment initiation in Table S2.

### 2.2 Treatment Response

We calculated treatment response as a dichotomous outcome, defined as a ≥ 50% reduction in symptom severity score from pre‐treatment to post‐treatment (Rush et al. 2006). Symptom severity scores were measured using one or more of the following scales: the Montgomery Asberg Depression Rating Scale (MADRS), the Hamilton Depression Rating Scale (HDRS), and the Beck Depression Inventory (BDI). When scores were available from more than one scale, we selected clinician‐administered (MADRS, HDRS) over self‐reported (BDI) scales and the MADRS over the HDRS (Carmody et al. 2006). See Table 1 for the scoring method used per cohort. Outcomes were collected between 4 and 12 weeks after the start of treatment, depending on the study design per participating site (Table 1).

### 2.3 Data Acquisition and Preprocessing

Structural T1‐weighted 3D brain MRI scans were obtained from all six sites and processed according to the ENIGMA protocols (http://enigma.ini.usc.edu/protocols/imaging‐protocols/). We used FreeSurfer software (Fischl 2012) to perform cortical parcellation. These parcellations were visually inspected and statistically evaluated for outliers as part of quality control. Table S3 details the MRI scanners, acquisition parameters, and the FreeSurfer version used per site. For our exploratory analyses, subcortical segmentations were also created with FreeSurfer, from which we used the volumetric measures.

### 2.4 Machine Learning Pipeline Configurations

The primary goal of this study was to predict pharmacotherapeutic treatment response based on cortical structural MRI‐derived predictors. The primary outcome of predictive performance was balanced accuracy (bAcc), defined as the mean of the sensitivity and specificity. For our secondary aim, we investigated whether predictive performance was affected by the configuration of the machine learning pipeline. To this end, we tested variations in the pipeline configuration in four ways, of which an overview is available in Table S4. First, we created different representations from the cortical structural data derived from FreeSurfer. Second, we tested if including clinical data in the models yielded a predictive performance better than chance. Third, we tested the accuracy of three different machine learning models of varying complexity. Lastly, we applied two different CV schemes. When each of these four pipeline configurations was under investigation, we fixed the other three to isolate the effect of the configuration of interest. Variations were fixed by either taking the average of all available options (for data representations and machine learning models) or by defining a default method (for clinical predictors and CV‐scheme). An overview of these options and the used defaults can be found in Table S4, the motivation of which can be found in the Supplementary Methods in Data S1.

#### 2.4.1 Data Representations

**FIGURE 2:**
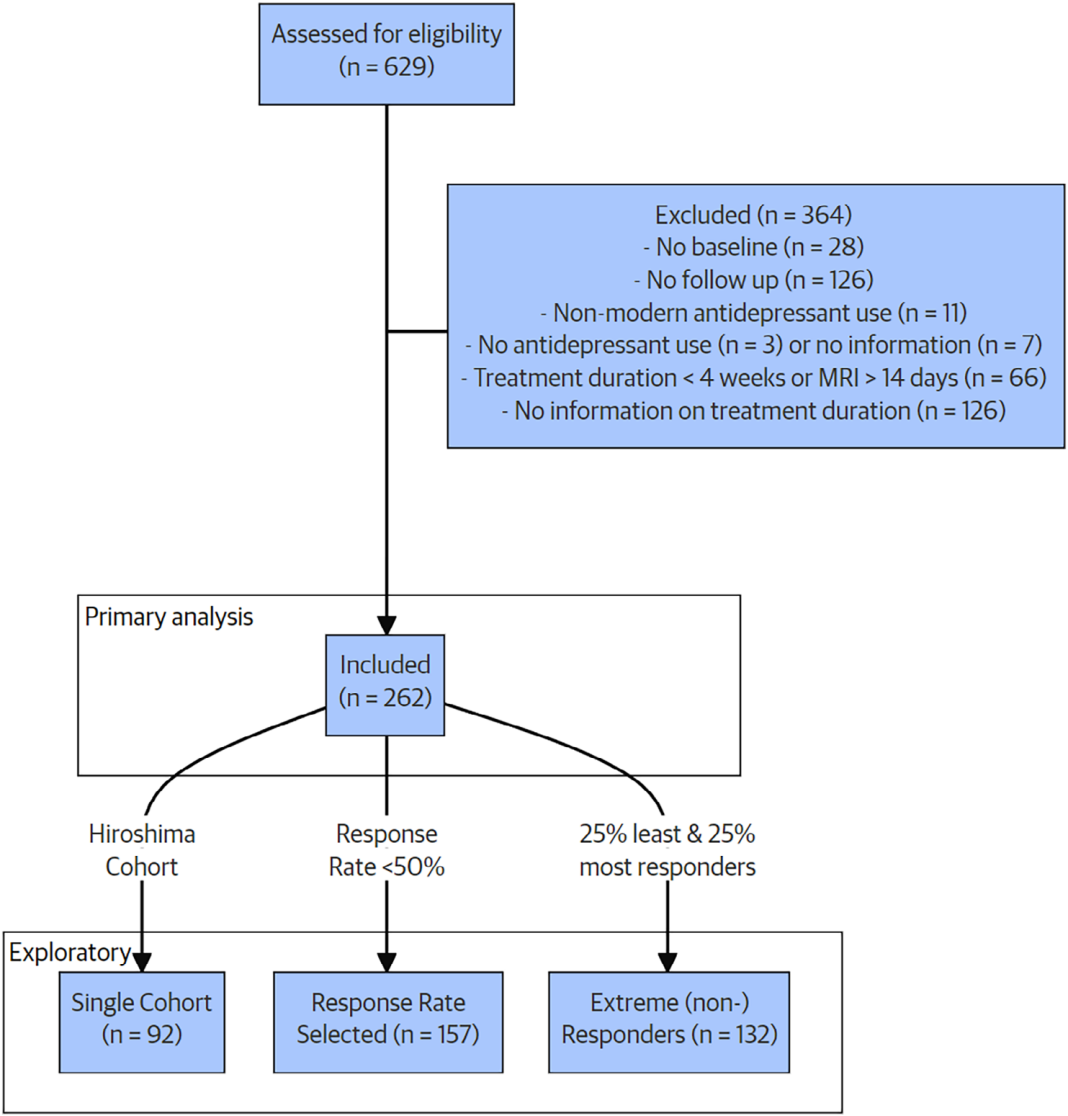
CONSORT flow diagram of patient inclusion.

FreeSurfer output consisted of a three‐dimensional mesh of the cortical surface of each hemisphere. Each mesh consists of 163 thousand vertices at which data was available on the cortical thickness, surface area, and Desikan–Killiany‐based (Desikan et al. 2006) gray matter regions of interest (ROI). We processed these data in three ways, resulting in three different cortical data representations (Figure 2). First, we averaged the cortical thickness and the surface area for each of the 34 ROIs per hemisphere, resulting in 136 predictors (a. ROI average). Second, we converted the voxel‐wise cortical surface area and cortical thickness measurements to a single one‐dimensional (1D) vector by downsampling using spatial linear interpolation, resulting in 900 predictors (b. cortical vector). Third, we created two other cortical data representations by projecting the cortical surface thickness (c. cortical thickness projection) and area (d. surface area projection) measurements to two‐dimensional (2D) planes of 64 × 64 pixels using stereographic projection (Su et al. 2013).

In addition to cortical data, subcortical volumes generated by FreeSurfer were available for seven subcortical regions. These regions were the nucleus accumbens, amygdala, caudate nucleus, hippocampus, globus pallidus, putamen, and thalamus. Volumes were available for both hemispheres. In addition, total intracerebral volume was available, which was used to normalize subcortical region volumes. Thus a total of 15 additional predictors was included in this exploratory analysis.

#### 2.4.2 Clinical Predictors

Clinical and demographic variables included as additional predictors were age, sex, age at first depressive episode, recurrence of MDD, antidepressant use at the time of scanning, and pre‐treatment symptom severity. When multiple symptom scorings were available, a single preferred score was used. The preferred score was the same as described in Section 2.2: preferring MADRS over HDRS, and HDRS over BDI. Symptom severity scores were normalized for each scoring instrument (Fang et al. 2020) by centering them at zero and scaling them to have a standard deviation of one for each specific scoring method across all cohorts.

#### 2.4.3 Machine Learning Model

To test different machine learning variations, we trained three types of models: (i) a Support Vector Classifier (SVC) implemented in SciKit‐Learn version 1.1.2; (ii) a Gradient Boosting Classifier (GBC) implemented in XGBoost (version 2.1.1) (Pedregosa et al. 2011) with key algorithm hyperparameters optimized using Bayesian optimization implemented in SciKit‐Optimize (version 0.10.2) for 25 iterations (see Supplementary Methods for details in Data S1), and (iii) a deep learning residual network (ResNet) implemented in PyTorch (v.1.13.0). These methods aim to capture both the most common, and more promising methods (Gao, Calhoun, and Sui 2018). The machine learning models were trained in the following setup.

For the SVC and GBC, the machine learning setup consisted of five steps: imputation, harmonization, feature selection, scaling, and classification. Each step in the pipeline was only fitted on data from the training partition to avoid information leakage from the testing partition. First, we imputed the few FreeSurfer values missing due to internal quality control using *K*‐nearest‐neighbor imputation (*K* = 5), as SVC does not support missing values. Second, we removed potential confounding effects of age and age^2^ using linear regression. Third, we used ComBat harmonization (Janssen, Mourao‐Miranda, and Schnack 2018) to mitigate confounding site effects. Covariates used in harmonization were age, age^2^, sex, and brain volume. Third, feature selection was performed using L1‐based feature selection. Fourth, we scaled feature values using *Z*‐score scaling to reduce scale sensitivity. Finally, we fitted the estimator.

The ResNet model was an 18‐layer ResNet (He et al. 2016), pre‐trained on ImageNet 1 K version 1 (Deng et al. 2009), with the final fully connected layer swapped with a 512 × 2 fully connected layer. We used the Adam optimization algorithm with a binary cross‐entropy loss implemented in PyTorch (Kingma and Ba 2014). Twenty percent of the training samples were held out as a validation set. Models were trained using a batch size of 32 for a minimum of 20 epochs, after which training was stopped if performance on the validation set ceased to improve for 10 epochs. The model with the lowest loss on the validation set was tested on the test set. Models were trained on GeForce RTC 2090 SUPER (NVIDIA Corporation, Santa Clara, California) for about 5 h.

#### 2.4.4 Cross‐Validation Methods

All machine learning models were trained in one of two CV methods implemented in SciKit‐Learn (v.1.1.2). The first method was outcome‐stratified k‐fold (SKF) CV, for 10 folds. The second method systematically excluded a single cohort from the training set to be used as a test set to assess the generalizability and robustness of our method across cohorts (leave‐one‐site‐out cross‐validation; LSO‐CV). These methods help distinguish between inherent variance and inter‐site variance in model performance.

### 2.5 Primary and Secondary Analyses

For our primary analysis, we tested if the mean accuracy of the models on the test set was statistically better than chance. Chance was defined as the prevalence of the majority response class, which was determined based on the training set. Whenever we tested accuracy against chance, we used permutation testing implemented in SciPy (v.1.7.3) with 100 permutations. *p* values were calculated using conservative approximation (Phipson and Smyth 2010; Ernst 2004). For model configurations found to be significantly better than chance, we report classifier feature importance using the coefficients of the SVC and impunity‐based feature importance in the GBC.

In the secondary analyses, we tested if there were significantly different mean performances among the four data representations, the inclusion of clinical predictors, three machine learning models, and two CV methods. For the cortical data representations and machine learning models, we performed a permutational multivariate analysis of variance test (PermANOVA) (Anderson 2005). For the inclusion of clinical data and the CV method, we compared the options using the permutation test mentioned earlier.

### 2.6 Exploratory Analyses

In the first post hoc exploratory analysis, we tested the accuracy of our machine learning model configurations on subpopulations with increased homogeneity in either the sample or in the dichotomous treatment response labels. For this purpose, we defined three subpopulations for which we repeated all our analyses steps described previously. In the first subpopulation analysis, we limited the sample to the single largest cohort to ascertain if inter‐cohort variance played a role in our prediction outcomes (a. single cohort). This subpopulation consisted of 92 patients from the Hiroshima cohort. Second, we repeated our analyses in cohorts with a mean response rate below 50% since the response rate varied substantially among cohorts (b. response rate selected cohorts). This subpopulation comprised 157 patients from the AFFDIS, Hiroshima, and Melbourne cohorts. Lastly, we created more homogeneous response outcome labels by defining a subpopulation consisting of the extreme subgroups of responders and non‐responders, that is, the 25% of patients showing the lowest percentage changes in depression severity and 25% responding the largest percentage changes to antidepressant treatment (c. extreme (non‐)responders). This subpopulation consisted of 132 patients (roughly equally distributed across cohorts). More demographic information about this subpopulation can be found in Table S5.

Finally, in our secondary exploratory analysis, we repeated our primary analysis but also included subcortical volumetric measures (only available for the ROI average data representation). We compared model performances between models that did or did not include subcortical data with the permutation test (see Supplementary Methods in Data S1 for the methodology used for this exploratory analysis).

## 3 Results

### 3.1 Population

Six international ENIGMA MDD Working Group samples contributed data to our analysis (Table 1). We received neuroimaging and clinical data from 629 participants with MDD. Following screening, 364 patients were excluded (for detailed information on the exclusion of patients, see the CONSORT flow diagram in Figure 1). Two of the main reasons for excluding patients were lack of follow‐up information (*n* = 126) and lack of information on treatment duration (*n* = 126). A total of 262 patients were included in the analyses. The mean age was 36.5 ± 15.3 years; 154 (59%) were female; the mean response rate was 57%; (Table 1; Figure 1). Two cohorts included adolescents (age < 20 years, Melbourne: 23/49 and Minnesota: 13/13 patients) with a considerably lower average age. Response rates varied substantially across sites (38%–84%) without clear differences between responders and non‐responders in treatment duration (8.7 ± 3.3 vs. 7.8 ± 3.1 weeks) or mean age as non‐responders (35.6 ± 15.0 vs. 37.6 ± 15.6 years, Table S6 for patient characteristics by treatment response). Response rates were high in SNRI users (78%, *n* = 52/66), as 83% of the SNRI users originated from the site that showed the highest response rate. Baseline clinical symptomatology was not predictive of treatment outcome at follow‐up (bAcc = 52.2% ± 8.5). FreeSurfer data for cortical thickness and surface area projections were available for 258/262 patients.

### 3.2 Treatment Response Performance

Overall, the performance in predicting antidepressant treatment response in MDD patients, using combined cortical thickness and surface area pre‐treatment from structural MRI data, was not significantly better than chance across machine learning pipeline configurations (bAcc = 50.5%; *p* = 0.66). These results were not different if we restricted our sample to subjects scanned < 7 days after start of treatment, nor influenced by treatment duration ( Tables S1 and S2).

### 3.3 Comparative Analyses of Machine Learning Pipeline Configurations

In secondary analyses, we first compared four types of cortical data representations (i.e., ROI average, cortical vector, and cortical thickness and surface area projections). None of the cortical data representations outperformed others (*p* = 0.10), and none of the cortical data representation performed significantly better than chance. Second, the additional inclusion of clinical data was examined. Performance of models that included clinical data (bAcc = 50.5%) did not outperform models without (bAcc = 51.0%; *p* = 0.70). In addition, none of these models significantly performed better than chance. Third, we compared three machine learning model types: SVC, GBC, and ResNet classifier. None of the models outperformed others (*p* = 0.15), and none of the models outperformed chance. Fourth, we assessed CV methods. LSO‐CV performance (bAcc = 52.3%) did not differ significantly from SKF‐CV (bAcc = 50.5%; *p* = 0.73). A complete overview of all outcomes and bAcc per model configuration is presented in Table 2.

**TABLE 2:** Main model performance for all research questions.

RQ1: Overall performance	Balanced Accuracy		Accuracy		Chance		Different from chance
	Mean	SD	Mean	SD	Mean	SD	*p*‐value
Full population	50.5	5.9	53.6	7.2	53.2	6.8	0.657
**RQ2‐I: Cortical data representations**							**0.101**
a. ROI average	50.6	5.4	54.6	5.6	54.6	5.1	0.612
b. Cortical vector	50.9	7.7	55.1	6.6	54.9	5.7	0.630
c. Cortical thickness projection	50.3	5.0	48.1	6.1	46.6	6.4	0.273
d. Surface area projection	49.8	2.1	53.9	9.1	53.7	8.2	0.436
**RQ2‐II: Adding clinical data**							**0.703**
Cortical data only	51.0	5.5	54.4	6.3	54.2	5.7	0.617
Clinical data added	50.5	5.9	53.6	7.2	53.2	6.8	0.657
**RQ2‐III: Machine learning model**							**0.153**
Support vector classifier	50.5	4.3	55.9	4.3	55.8	3.8	0.545
Gradient boosting classifier	51.0	8.3	53.8	7.4	53.7	6.5	0.701
ResNet	50.0	3.9	51.0	8.3	50.1	8.2	0.372
**RQ2‐IV: Cross‐validation method**							**0.727**
10‐Fold cross‐validation	50.5	5.9	53.6	7.2	53.2	6.8	0.657
Leave‐site‐out cross‐validation	52.3	5.5	51.5	10.4	48.7	11.5	0.235
**Exploratory I: Subgroup performance**							**0.002**
Single cohort	49.6	7.1	48.4	8.0	47.5	10.6	0.666
Response rate selected cohorts	50.1	7.2	52.1	10.0	52.1	9.3	0.732
Extreme (non‐)responders	63.9	10.6	63.6	8.7	52.1	10.3	0.001
**Exploratory II: Adding subcortical data**							**0.703**
Cortical data only	51.6	3.7	55.3	3.8	55.4	5.7	0.587
Subcortical data added	50.5	5.9	53.6	7.2	53.2	6.8	0.657

*Note:* The balanced accuracy, accuracy, and priori chance are provided for each machine learning pipeline configuration investigated. The *p*‐value is provided on the right, which illustrates whether a machine learning pipeline configuration outperforms chance. The overarching *p*‐values for each research question express the probability of significantly different mean performance among (using permutational multivariate ANOVA) or between (permutation test) the configuration variations tested, for example, for RQ2‐II whether the balanced accuracy of “Cortical data only” differs significantly from “Clinical data added,” tested using the permutation test. Significance was inferred when *p* < 0.05.

Abbreviations: ResNet, deep learning residual network; ROI, region of interest; RQ, research question.

### 3.4 Exploratory Subpopulation Analyses

**FIGURE 3:**
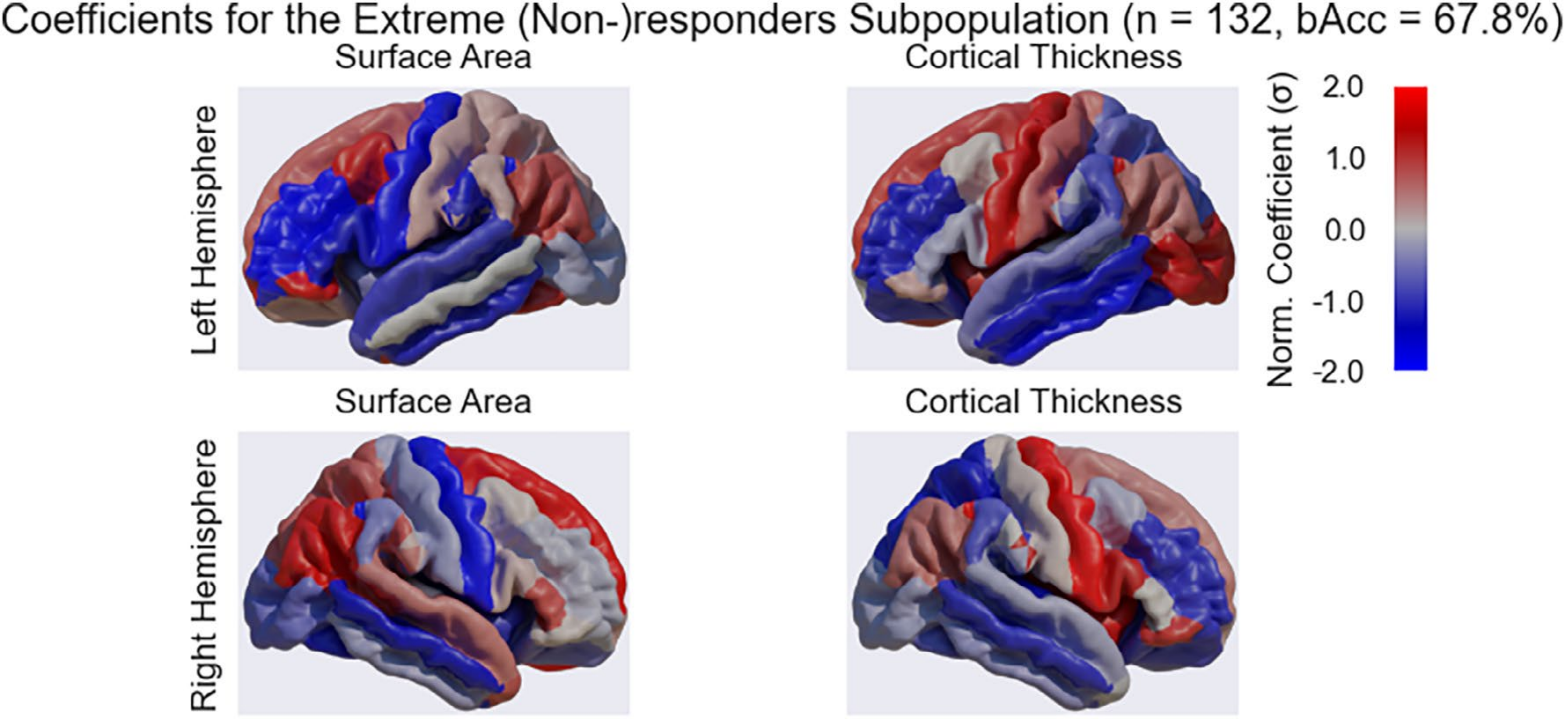
Region of interest relevance for the exploratory analyses. The figure shows the normalized coefficients used by a machine learning model to predict treatment response in the extreme (non‐)responders subpopulation, overlaid on a standard structural brain (cortical data representation: ROI average; model: gradient boosting classifier [GBC]; only cortical data). The sign indicates the direction of the relationship between a positive treatment response and either surface area (left two panels) or cortical thickness (right two panels). Red indicates a positive direction, whilst blue indicates a negative direction. The magnitude (visualized as saturation) of the coefficients indicates the strength of the relationship.

We conducted exploratory subpopulation analyses to evaluate the performance of the models in three different subpopulations. Neither the performance of the single cohort subpopulation (bAcc = 49.6%; *p* = 0.67) nor of the response rate selected subpopulation (bAcc = 50.1%; *p* = 0.73) outperformed chance across pipeline configurations. In the extreme (non‐)responders subpopulation, our models did perform significantly better than chance (bAcc = 63.9%; *p* = 0.001). All outcomes per machine learning pipeline configuration for this subpopulation are provided in Table S7. In short, all pipeline configurations we tested for this subpopulation significantly outperformed chance. BAcc did not improve significantly when clinical or subcortical data was added, or when LSO‐CV was applied. Predictors contributing most to this prediction were bilateral increased cortical thickness in the precentral gyri, smaller surface area of the precentral gyri, and larger surface area of the superior frontal gyri (Figure 3). All coefficients are provided in Table S8.

Finally, we tested whether addition of subcortical predictors improved predictive performance. Performance of models including subcortical data (bAcc = 51.6%) was not significantly better than models without subcortical data (bAcc = 50.5; *p* = 0.31). Again, our models only performed significantly better than chance in the extreme (non‐)responders subpopulation. All outcomes of this analysis, and of alternative classifiers are provided in Tables S9, and S10, respectively.

## 4 Discussion

The present study aimed to investigate machine learning based classification approaches using pre‐treatment cortical structural MRI‐derived measures to predict antidepressant treatment response. Using the largest sample of cortical structural MRI data to date (Cohen et al. 2021; Lee et al. 2018), our work presents compelling evidence that, using common machine learning approaches, cortical thickness and surface area alone are not viable biomarkers for predicting antidepressant treatment response in individuals with MDD. This finding was independent of machine learning pipeline configurations concerning (I) cortical data representations, (II) inclusion of clinical data, (III) machine learning models, and (IV) cross‐validation methods. Moreover, our exploratory subpopulation analyses highlight the possible viability of employing cortical structural measures as predictive markers for antidepressant treatment response in patients with MDD, elucidating specific patterns linked to the highest and lowest responsive individuals only.

### 4.1 Overall Prediction of Treatment Response

In contrast to our primary hypothesis, we could not predict treatment response in MDD better than chance level. Although several prior studies highlighted associations between baseline cortical thickness—in, for example, the anterior cingulate cortex (Phillips et al. 2015), supplementary motor area (Wu et al. 2021), and insula (Pimontel et al. 2021)—and pharmacological treatment response or remission in patients with MDD, we were unable to provide support for this framework. However, in line with our findings, another similar study also reported very limited value in using baseline structural MRI to predict pharmacological treatment outcome in MDD (Beliveau et al. 2022). Moreover, a large multi‐center study also reported no differences in pre‐treatment or follow‐up cortical thickness between responders and non‐responders (Suh et al. 2020). These divergent findings underscore the possibly complex relationship between cortical thickness and treatment response in MDD, which is further illustrated by a whole‐brain imaging study demonstrating intricate patterns of cortical and subcortical regions involved in the prediction of remission and residual symptoms in MDD (Costafreda et al. 2009). However, as these previous studies investigated associations with treatment response at the group level and not at the individual level, our findings cannot directly be compared to these previous findings. In addition, although subcortical regions such as the hippocampus have previously been suggested as a strong predictor for antidepressant treatment outcomes (Colle et al. 2018; Fu, Steiner, and Costafreda 2013; Hu et al. 2019; MacQueen et al. 2008), the inclusion of subcortical data in our exploratory analyses did not significantly alter model performance. However, it should be noted that only volumetric measures were available for subcortical data. This prevented the use of more sophisticated machine learning models (e.g., deep learning residual network) for the individualized pharmacotherapeutic treatment response predictions and, therefore, these findings should be interpreted with caution.

### 4.2 Effect of Model Configurations on Performance

For our second hypothesis, we tested several common machine learning pipeline configurations. Contrary to our expectations, we generally did not find a significant performance difference when including clinical data, nor for more complex data representations and different machine learning models. One outlier was a significant difference in performance for different data representations, but performance here was still not significantly better than chance level. Similarly, other studies suggest that deep learning does not consistently outperform classical machine learning when applied to high‐dimensional data and relatively low sample sizes (Sajjadian et al. 2021; Squarcina et al. 2021).

Our results do not demonstrate an association between baseline clinical symptomatology and treatment outcome. This is in accordance with some (Friedman et al. 2012; Klein, Shankman, and Rose 2008) but in contrast to other (Perlman et al. 2019; Uher et al. 2012) previous works. This heterogeneity in findings is one of the reasons why the on‐going search for additional biomarkers for treatment response remains so relevant to date (Rost, Binder, and Brückl 2023). As prior studies by Rajpurkar et al. (2020) and Poirot et al. (2024) have shown that symptomatological predictors can be integrated with machine learning to improve predictive power, we additionally included clinical symptomatology to our machine learning approach. However, we found that including these predictors did not significantly boost model performance. However, our results are severely limited by the lack of models that performed well, which reduces any effect that can be expected in the comparisons we have made between machine learning models. This also limits the generalizability of these findings to other predictive studies. At the same time, it provides thorough evidence to researchers that cortical structural properties may contain limited information and alternative approaches such as other MRI sequences (Squarcina et al. 2021), multimodal data (Sajjadian et al. 2021) or alternative analysis methods such as normative modeling (Rutherford et al. 2023) may prove more fruitful.

### 4.3 Exploratory Subpopulation Analyses

Our exploratory analyses suggest that predicting pharmacotherapeutic treatment response on an individual level is only feasible when limiting the sample using a variable that is defined at the outcome level. Here, we selected a subpopulation of patients with MDD exhibiting either the least or the most improvement in clinical symptoms following pharmacological treatment. Therefore, we advise caution in interpreting these findings as we selectively examined a particularly distinct subpopulation with limited generalizability to a clinical population. Our findings seem to suggest the presence of distinct populations within the MDD population, based on the association between cortical structural MRI and treatment outcome. These results appear to indicate the presence of biologically distinct response‐related subpopulations within MDD. Supporting this, the most influential predictors within this subgroup are cortical structural features rather than clinical variables. As response prediction was only feasible for individuals showing the most and least improvement in symptoms, it is possible that antidepressant response in MDD cannot readily be characterized using a continuous spectrum. A similar observation was made in a study by Deserno et al. (2022), who investigated phenotypic clustering based on questionnaire data in a combined sample of attention–deficit hyperactivity disorder and autism spectrum disorder populations. They similarly found only clear clusters of more extreme symptom‐related subgroups, but no clear segregation between the other individuals. However, the extent and underlying mechanisms of potential biological differences in MDD subpopulations remain unclear and further research is needed to elucidate these aspects.

The predictors that appear to contribute most to the prediction of treatment response in the extreme (non‐)responders subpopulation were located in the precentral gyri (higher cortical thickness and smaller surface area) and superior frontal gyri (larger surface area). Interestingly, prior studies have shown that individuals with MDD tend to have lower cortical thickness in the precentral gyrus and lower gray matter volumes compared to healthy volunteers (Xiong et al. 2019; Zhang et al. 2012). Prior studies have also highlighted alterations in gray matter volume (Lai and Wu 2014) and functional connectivity (Yang et al. 2017; Zhu et al. 2022) of the superior frontal gyrus in individuals with MDD compared to healthy controls. A systematic review by (Porta‐Casteràs et al. 2021) also recently reported increased functional connectivity of the superior frontal and middle frontal gyri following electroconvulsive therapy. Taken together, the brain regions contributing most to the model may tentatively be implicated in the pathophysiology of MDD and altered following treatment, although the underlying mechanisms involved are not yet understood.

### 4.4 Strengths and Limitations

We performed the largest mega‐analysis to date, using data from six cohorts from the ENIGMA MDD consortium, the largest consortium for MDD neuroimaging research (Dinga et al. 2018). The homogeneity of measurement strategies across settings is of paramount importance for prediction research (Luijken et al. 2019). Preprocessing of the structural data was standardized across all participating sites. Crucially, a previous ML study using data from the ENIGMA‐MDD consortium showed that remaining site‐effects may still affect classification outcomes (Belov et al. 2022). Therefore, we used ComBat to remove remaining site‐effects in a step called harmonization. Since site differences that are not modeled explicitly are regressed out as a site‐effect, harmonization potentially reduces the signal‐to‐noise ratio (Orlhac et al. 2022). In our exploratory analyses, we further investigated the effect of heterogeneity in the sample by selecting more homogeneous subpopulations and testing SKF‐CV against LSO‐CV. Model performance for these subpopulations did not increase significantly, and LSO‐performance was comparable to SKF‐CV, suggesting that this heterogeneity is not the main factor contributing to the difficulty of predicting treatment response using cortical MRI predictors.

In our mega‐analysis approach, patients were drawn from various international cohorts. Smaller and more homogeneous samples run the risk of overfitting and reduced out‐of‐sample performance, as noted in previous studies (Bracher‐Smith, Crawford, and Escott‐Price 2021). Notably, reviews by Sajjadian et al. (2021) and Flint et al. (2021) report an inverse correlation between sample size and accuracy, with smaller samples tending to yield higher reported accuracies. Both decreased overfitting and increased homogeneity in the current sample may account for the low performance observed in the present study compared to previous studies reviewed by Squarcina et al. (2021). This discrepancy underscores the importance of sample size and diversity considerations, as well as standardized protocols, to reliably evaluate the performance of machine learning models for personalized medicine.

Our analyses were pre‐registered and approved by the ENIGMA MDD consortium. By pre‐registering our analyses before receiving the data, we avoid inadvertently tuning our analyses to our data (Smith and Ebrahim 2002). However, this preregistration also limited the scope of this work to analyses of cortical structural data in the entire population. To retain a clear distinction between pre‐registered and post hoc analyses, while expanding the predefined scope with additional analyses, we explicitly report non‐preregistered analyses as exploratory.

As an ENIGMA MDD mega‐analysis on the prediction of individual pharmacological treatment response, major strengths of our investigation include the large sample size, the preregistered analyses, and the use of various machine learning pipeline configurations. The sample size specifically, is on the higher end of the field (Sajjadian et al. 2021) and within the estimated range of the number of required samples of 100–300 (Beleites et al. 2013; Luedtke, Sadikova, and Kessler 2019). However, drawbacks remain. We were unable to investigate whether the inclusion of additional MRI modalities contributes to model performance, as this data was not available for the current study. In addition, we included pre‐existing data from international populations, which means that study design and data collection techniques were not standardized across sites. Many factors, including for example the inclusion criteria, the timing of pre‐treatment MRI scans, treatment duration, use of additional medication, timepoints during which response is determined, and study design (naturalistic follow‐up vs. clinical trials), remained unstandardized across the included sites. In addition, further heterogeneity may be introduced by variation in cultural variation in the clinical presentation of depression (Kirmayer 2001) and the clinical assessment of depression symptom severity (e.g., inter‐rater variability and diagnostic tools used per site). It should be acknowledged that these sources of variation may have a considerable effect on model performance and the outcomes of our mega‐analysis. In addition, it is currently unclear how the choices of MRI processing impact performance of prediction models. It is possible that, in this study, possibly clinically relevant features in cortical structural MRI data are omitted by the (pre‐)processing and dimensionality reduction choices made.

### 4.5 Future Directions

While this study used a single imaging modality to develop personalized predictions of treatment response to antidepressant medication, incorporating multiple predictors across modalities may increase accuracy. A meta‐analysis by Lee et al. (2018) found that combined or multi‐modal predictors performed better than any single modality alone, which was recently corroborated by a similar machine‐learning based prediction study for pharmacological treatment outcome in depression (Poirot et al. 2024). Although accuracy in our study did not improve when adding clinical data, another study that focused solely on clinical and behavioral predictors was able to predict pharmacological treatment response with high accuracy (Zhou et al. 2022). In addition, Poirot et al. (2024) reported that including clinical data to multi‐modal MRI‐based predictors boosted model performance. These results suggest that future research may benefit from using machine learning algorithms incorporating multimodal data to predict pharmacotherapeutic treatment response in individuals with MDD.

Another method that may boost model performance is to obtain early change information (e.g., after 1 or 2 weeks of treatment) in cortical structural MRI‐derived measures for predictions later in treatment, or to consider including multiple time points. A study by Bartlett et al. (2018) observed that the change in anterior cingulate cortical thickness during the first week of treatment predicts treatment response to SSRIs. Harris et al. (2022) also showed that models using MRI predictors obtained both pre‐treatment and 2 weeks after treatment initiation outperformed models using one of these measures. Since this data was unavailable, we were unable to assess the effect of adding the information to the analyses in the current study.

Encouraging findings have also emerged from studies exploring alternative approaches. For example, studies considering heterogeneity in symptom profiles, for example assessed with psychometric network modeling, have shown distinctions in symptom network structure between remitters and non‐remitters following pharmacotherapeutic treatment (van Borkulo et al. 2015). This highlights the potential of these methods for unraveling interindividual variations in depression symptom presentation in relation to treatment outcome. Alternatively, a recent study using normative models of structural MRI measures and brain functional connectivity demonstrated promising results in group difference testing and classification tasks (Rutherford et al. 2023). Although the application of normative modeling for complex classifications such as individual pharmacological response prediction has not been evaluated, further investigations of this method's efficacy may prove fruitful.

## 5 Conclusion

In this mega‐analysis of cortical structural MRI in 265 individuals with MDD based on data from the ENIGMA‐MDD cohort, we provide compelling evidence that cortical structural MRI alone is not a reliable predictor of individualized pharmacotherapeutic treatment response in MDD. This finding was observed consistently across the machine learning pipeline configurations we employed, which included the majority of predictive methods commonly covered in the literature. Specifically, we varied the cortical data representation, the inclusion of clinical data, machine learning model, and CV scheme. Findings from our exploratory subpopulation analyses, however, suggest the potential of cortical structural measures, alone or combined with subcortical volumetric measures, in predicting antidepressant treatment response for MDD patients, linking distinct patterns to the most and least responsive individuals. To improve the accuracy of personalized treatment response prediction, we suggest further evaluation of alternative approaches, such as integrating multiple imaging modalities.

## Conflicts of Interest

M. W. A. Caan is a shareholder of Nico‐lab International Ltd. Dr. H. G. Ruhe received speaking fees from Lundbeck and Janssen, and grants from ZonMW, Hersenstichting, the Dutch ministry of health and an unrestricted educational grant from Janssen. All other authors declare no financial relationships with commercial interests.

## Supporting information


**Data S1:** Supporting Information.

## Data Availability

Data sharing is not applicable to this article as no new data were created or analyzed in this study.
