# Correction to “Predicting Antidepressant Treatment Response From Cortical Structure on MRI: A Mega‐Analysis From the ENIGMA‐MDD Working Group”

**DOI:** 10.1002/hbm.70144

**Published:** 2025-02-05

Poirot, M. G., D. E. Boucherie, M. W. A. Caan, et al. 2025. “Predicting Antidepressant Treatment Response From Cortical Structure on MRI: A Mega‐Analysis From the ENIGMA‐MDD Working Group.” *Human Brain Mapping* 46, no. 1: e70053. https://doi.org/10.1002/hbm.70053.

The ITEA (Reference number 21016 DAIsy) funding program was missing from the Acknowledgments section in the originally published version. Here is the corrected text:

This work was done with the ENIGMA major depressive disorder (ENIGMA‐MDD) working group and was supported by the Eurostars (Reference number 113351 DEPREDICT) and ITEA (Reference number 21016 DAIsy) funding programs.

The online version has been corrected accordingly.

We apologize for this error.

