# Supplementary material for: Predicting Antidepressant Treatment Response From Cortical Structure on MRI: A Mega‐Analysis From the ENIGMA‐MDD Working Group
Source: Hum Brain Mapp. 2025 Jan 6;46(1):e70053. doi: 10.1002/hbm.70053 (PMC11702469; doi:10.1002/hbm.70053)
Supplement: Supplementary file 1 — Data S1: Supporting Information. [file HBM-46-e70053-s001.docx]

| Supplementary Materials  Poirot MG, Boucherie DE, Caan MWA, et al. Predicting Antidepressant Treatment Response from Cortical Structure on MRI: A Mega-Analysis from the ENIGMA MDD Working Group  **Content**  [Supplementary Methods 1](#_Toc166243825)  [Accepted Analysis Plan 1](#_Toc166243826)  [Table S1. Performance for Long Treated Patients Compared to the Whole Population. 12](#_Toc166243827)  [Table S2. Sensitivity Analysis for Period between Baseline MRI andTreatment Initiation 13](#_Toc166243828)  [Table S3. Information on the MRI Scanners and Acquisition Protocols Used for Each Cohort. 14](#_Toc166243829)  [Table S4. Pipeline Configuration Variations and Defaults 15](#_Toc166243830)  [Table S5. Patient Characteristics of the Extreme (Non-)responders Subpopulation. 16](#_Toc166243831)  [Table S6. Patient Characteristics by Treatment Outcome. 17](#_Toc166243832)  [Table S7. Performance for the Exploratory Analysis of the Extreme (Non-)responders Subpopulation. 18](#_Toc166243833)  [Table S8. Feature Contributions in the Subpopulation of Extreme (Non-)responders 19](#_Toc166243834)  [Table S9. Model Performance for Exploratory Analyses of the Inclusion of Subcortical Data. 20](#_Toc166243835)  [Table S10. Exploratory Analysis using Random Forests 21](#_Toc166243836) |
| --- |

# Supplementary Methods

# Accepted Analysis Plan

The following is section 2 of Appendix A, containing the Analysis Plan, as accepted by the Enhancing Neuro Imaging Genetics through Meta-Analysis Consortium (ENIGMA) – Major Depressive Disorder Working Group, as accepted by March 2021.

**2.Analysis Plan**

**Proposal Title:**

Structural Imaging predictors of antidepressant treatment response in the ENIGMA-MDD working group.

**Give a brief summary of your analysis plan that includes the following:**

§ Research questions

Main Research Question:

1. Can a machine learning algorithm –using deep learning and radiomics– predict antidepressant (AD) treatment response in individuals with major depressive disorder (MDD) using structural brain MRI measures?
   *Hypothesis 1:* machine learning based classification using structural MRI-derived measures can adequately predict AD treatment response.
2. Which (set of) (clinical and/or imaging) features have the largest contribution to the predictive method in our main research question?
   *Hypothesis 2:* Specific imaging (e.g. prefrontal cortex shape and/or volume) and clinical (e.g. MDD disease severity) features can be identified as having the largest contribution in predicting AD treatment response in individuals with MDD

**§ Brief background and rationale for addressing the research question within ENIGMA MDD**

Previous successes of the ENIGMA MDD consortium have provided novel insights on alterations of cortical and subcortical structures, subcortical shape alterations, white matter disturbances and structural asymmetry, through some of the largest MDD-related collaborative projects in the world (de Kovel et al. 2019; Schmaal, Hibar, et al. 2016; Schmaal, Veltman, et al. 2016; van Velzen et al. 2020). By showing case-control differences, these works have laid the groundwork for future translational research. Our group is currently working to translate such findings into a tool for AD treatment response prediction. Reducing unsuccessful treatment trials could improve depression treatment. Additionally, insights into this algorithm will improve the understanding of manifestations of treatment response in medical imaging. We propose to initiate a large-scale investigation into imaging brain-MRI biomarkers correlated to treatment response.

Currently, AD treatment planning follows a trial-and-error strategy, with only a third of MDD patients showing significant symptom relief in response to the initial treatment (Rush et al. 2009) and for 50% of patients an efficacious AD has not been found after one year (Keitner et al. 1992; Rush 2007). To shorten this lengthy process, algorithms for treatment response prediction are needed. These algorithms will be developed using data driven methods such as radiomics –the extraction of large numbers of features from medical images– and statistics, such as machine learning and deep learning. Data of large existing, external data sets consisting of magnetic resonance imaging (MRI) measurements, clinical assessments and patient health information are extremely valuable to pursue this goal.

Previous work on morphometric changes for AD treatment response has identified several subcortical structural changes, summarized in work by Schrantee et al. (Schrantee, Ruhe, and Reneman 2020). For instance, meta-analyses have shown that a smaller right hippocampal volume was a significant predictor of poorer treatment response in MDD (Colle et al. 2018; MacQueen et al. 2008; Vythilingam et al. 2004). In addition, larger tail and subiculum volumes have been shown to be predictive of symptom reduction (Hu et al. 2019; Maller et al. 2018). Cortically, non-responding patients exhibited thinner cortex in left rostral middle frontal cortex at baseline (Suh et al. 2020).

A major challenge in translating these potential predictive biomarkers into the clinic are the generally underpowered studies with a large amount of variation in techniques and analysis approaches (Schrantee, Ruhe, and Reneman 2020). To overcome this challenge a collaborative approach is warranted (Fonseka, MacQueen, and Kennedy 2018).

Our main goal in this ENIGMA MDD mega-analysis is to develop and validate an algorithm capable of predicting treatment response based on individual FreeSurfer derived imaging features. As to the potential performance of such a method, a recent review on deep learning for the prediction of treatment response in MDD shows that deep learning based methods outperform regression models, with relatively high area-under-the-curves (AUCs) ranging from 0.69 to 0.80 (Squarcina et al. 2021). This work also states that the major limitation on these findings remains their sample size. A different review on quantitative electroencephalography based response prediction in MDD has shown sensitivities of 0.72, specificities of 0.68 and an AUC of 0.76 to be achievable. However the authors do note substantial publication bias (Widge et al. 2019). The performance aimed for in this proposed study is therefore to be on par with existing literature, but to provide increased confidence in the methods generalizability due to the larger, more diverse sample.

The secondary objective is to elucidate the contribution of specific features to this prediction, to provide some mechanistic insight to the black box in nature of these methods using regression analysis.

Data wise, the focus will be on existing vertex data of cortical, and FreeSurfer output of subcortical areas, further specified in the next paragraph. Vertex data will provide us with first and second order morphological statistical features. Follow-up treatment response information should be available for participation, no further generation of data should be necessary.

**§ Variables to be used in the analysis** (the main predictor and outcome variables, and potential covariates must be identified)

Outcome variables:

- Information of AD treatment response at baseline and follow-up.
   These will be derived using scores on the individual items of the depressive symptom questionnaires (BDI scores & HDRS, or else if available also MADRS and/or (Q)IDS scores in *Covariates.csv* file)

Preferably we receive individual treatment outcome of the above depression severeity scales at baseline and 8 week follow-up . If this is not exactly what has been used in different studies we request the data most proximate to week 8 (and an indication of which week of treatment this dta concerns). Alternatively, if raw scores can somehow not be provided, change data (as %change of baseline score over time) will be considered.

Predictor measure:

- Individual’s Cortical FreeSurfer vertex mappings (*rh.area, rh.thickness* and if available *rh.curv*,*rh.volume*, *rh.sulc, rh.pial* and *rh.jacobian_white* from FreeSurfer output folder, registered to fsaverage space) and ROIs (*SurfAvg.csv*, *ThickAvg.csv*).
- Individual’s Subcortical FreeSurfer segmentation table values (aseg.csv, *LandRvolumes.csv*)

Clinical descriptors in exploratory analyses, validation and stratification, all from *Covariates.csv* file, with the exception of type of treatment intervention, which is yet to be formally collected:

- MDD status
- Age
- Sex
- Recurrence (first vs. recurrent episodes)
- Remission status
- Scan site
- Age of onset
- Severity (BDI, HDRS, MADRS,QIDS scores)
- AD medication use
- Intracranial Volume (ICV)
- Type of treatment intervention during follow-up

*ad 1) What data is required in addition to what is already available in the database (e.g. additional variables in the Covariates.csv file, new or additional imaging measures, etc?)*

Vertex-wise cortical surface area and thickness and maps have been collected as part of another project (Schmaal c.s.) but are not yet available from all sites. ROI FreeSurfer measures (subcortical volumes, cortical thickness, and surface area csv files), as well as covariates are already available within ENIGMA-MDD, with the exception of information on type of treatment intervention during follow-up.

**§ Level of data sharing**

*Please specify what level of data sharing is required. E.g., does the project involve raw scans, FreeSurfer (or other software or clinical) derived individual measures, group-level summary statistics, post-estimation statistics?*

Mega-analyses will be performed for this project on clinical and imaging derived information. No raw scans are required. Freesurfer derived individual measures and vertex-wise maps (as defined above) at the level of individual participants are required for this proposal.

*ad 1) In case sharing of raw scans or individuals measures is required for e.g. a* ***mega****-analytical statistical framework; please indicate why a* ***meta****-analytical approach using group-level summary statistics or post-estimation statistics is inadequate to answer the research question.*

We do not request individual’s raw scans. The goals of this proposed research are predictions for single individuals. We also aim to compute radiomics features on the vertex mapping and train a machine-learning algorithm on these data. This makes our approach incompatible with an approach using only group-level summary statistics. The question therefore does require individual measures to be collected.

*ad 2) In case sharing of raw scans or individuals measures is required for e.g. a* ***mega****-analytical statistical framework, please indicate where those scans and/or individual data will reside during the course of the project.*

During the course of the project, all data relating to the project will reside on our secured proprietary premise to which access is restricted to individuals mentioned below.

**§ Outline of analysis per research question**

**Aim 1:** To predict AD treatment response in individuals with MDD using structural brain MRI measures.

For aim 1, we will use supervised machine learning to build predictive models. Supervisory labels consist of treatment outcome defined as binary, with positive response defined as a larger than 50% reduction in symptom severity scores from baseline to follow-up as a percentage of baseline score (Lam et al. 2016). Outcome is classification accuracy, specified as specificity, sensitivity and area under the curve. The algorithm is deemed successful if performance metrics are at least on par with state of the art as mentioned earlier. We start with sparser models before including all covariates.

The prediction pipeline consists of subsequent feature generation, feature selection and classification. Input consists of vertex-wise mappings, corrected for site effects using ComBat. Feature generation consists of radiomics analysis of scalar overlays. From these overlays, local and global features can be derived, for example global mean cortical thickness. These cortical features are combined with subcortical FreeSurfer segmentation output.

Feature Selection methods used will be:

1. Least absolute shrinkage and selection operator (LASSO) regression
2. Ridge logistic regression
3. Elastic net logistic regression

Classifiers used will be:

1. Support Vector Machine (SVM)
2. Gradient Boosting
3. Neural network

We will explore if addition of demographic, behavioral and clinical information improves the performance of predictive models. Depending on the availability of these measures, these include age, sex, age of onset and illness stage. These data will also be used for stratification of cross validation folds and performance validation.

**Aim 2:** To identify which (set of) (clinical and/or imaging) features have the largest contribution to our predictive method in our main research question?

For aim 2, regression analyses will be performed on previously radiomic features described at aim 1. Label data-derived features will serve as dependent variables and treatment response as independent variables.

Covariates included are sex, age, recurrence status, remission status, age of onset, severity of depressive symptoms and type of treatment intervention during follow-up at time of scan. We are specifically interested in independent variables that generalize across cohorts. If the number of studies/subjects allow it, we will pool data per treatment type. Moreover, we will group different types according to drug class (e.g. SSRI, SNRI, NDRI, NaSSA, etc.). Depending on the variety of available response data, our initial intention is to use the 8 week outcome or else the nearest timepoint for primary analyses. If response shows strong correlation to duration of follow-up and this differs significantly among cohorts, we opt for correction if feasible or to separate the analyses.

Main outcomes are expressed as statistical correlation to the independent variable given a dependent variable. Group differences for responders versus non-responders are also analyzed following the same definition as for aim 1.

Data Preparation

*Imputation:* Within each study/dataset, missing values will generally be assumed to be missing at random (MAR), pending evaluation of bias (missing not at random, MNAR). Variables assumed to be MAR will be imputed by means of mean imputation, and variables assumed to be MNAR will be handled using a method called “missing incorporated in attribute” (Josse et al. 2020). Imputation will be performed on the level of radiomics features.

*Partitioning:* For aim 1, the cohort will be further partitioned into a training, validation and test set, in a 75/15/10% ratio respectively. In the training process, training data is used to train the model and validation data to inquire the learning progress on unseen data. Finally, test data is used evaluate the final model.

*Batch harmonization:* ENIGMA MDD brain images have been acquired across different scan sites. These sites use different scanners, different sequences, different durations of treatment and different types of antidepressants and are imbalanced with respect to age, sex, race and clinical status. These factors can cause batch effects, resulting in underperforming models. Simply including ‘scanner type’ as a confounding variable may not work well (Rao, Monteiro, & Mourao-Miranda, 2017). We will apply ComBat (Johnson, Li, and Rabinovic 2007), which has been shown to effectively reduce scanner-to-scanner variability while preserving biological associations (Beer et al. 2020; Fortin et al. 2018). In the proposed study, we will first apply ComBat on the vertex-wise maps to remove site-induced variances.

Analysis pipeline

*Cross validation:* All analyses will be carried out within a strict cross validation (CV) framework to assess generalizability of the performance estimation. Exhaustive CV will be performed in ten stratified folds. Covariates considered in stratification will be divided optimally for all folds a priori. To further strengthen generalizability of our results we will perform leave-site-out cross-validation (Koutsouleris et al. 2016) depending on the availability of data.

*Stratification:* Non-clinical covariates considered in stratification to represent the balance distribution in the main data set are age, site of scanning, sex and ICV (if this remains unbalanced after stratifying for sex). Clinical covariates are avoided in stratification to avoid class balance bias.

**§ References**

Beer, J. C., Tustison, N. J., Cook, P. A., Davatzikos, C., Sheline, Y. I., Shinohara, R. T., & Bassett, S. S. (2020). Longitudinal ComBat: A method for harmonizing longitudinal multi-scanner imaging data. NeuroImage, 220, 117129. https://doi.org/10.1016/j.neuroimage.2020.117129

Colle, R., Dupong, I., Moulier, V., Cury, C., Birmes, P., Bonafe, A., ... & Schmitt, L. (2018). Smaller hippocampal volumes predict lower antidepressant response/remission rates in depressed patients: A meta-analysis. World Journal of Biological Psychiatry, 19(5), 360–367. https://doi.org/10.1080/15622975.2016.1268712

Fonseka, T. M., MacQueen, G. M., & Kennedy, S. H. (2018). Neuroimaging biomarkers as predictors of treatment outcome in major depressive disorder. Journal of Affective Disorders, 233, 21–35. https://doi.org/10.1016/j.jad.2017.09.049

Fortin, J.-P., Cullen, N., Sheline, Y. I., Taylor, W. D., Aselcioglu, I., Cook, P. A., ... & Shinohara, R. T. (2018). Harmonization of cortical thickness measurements across scanners and sites. NeuroImage, 167, 104–120. https://doi.org/10.1016/j.neuroimage.2017.11.024

Hu, X., Ji, G.-J., Wang, K., Zhang, H., He, Y., & Gong, Q. (2019). Abnormal hippocampal subfields may be potential predictors of worse early response to antidepressant treatment in drug-naïve patients with major depressive disorder. Journal of Magnetic Resonance Imaging, 49(6), 1760–1768. https://doi.org/10.1002/jmri.26522

Johnson, W. E., Li, C., & Rabinovic, A. (2007). Adjusting batch effects in microarray expression data using empirical Bayes methods. Biostatistics, 8(1), 118–127. https://doi.org/10.1093/biostatistics/kxj037

Josse, J., Prost, N., Scornet, E., & Varoquaux, G. (2020). On the consistency of supervised learning with missing values. arXiv preprint arXiv:2002.12072.

Keitner, G. I., Ryan, C. E., Miller, I. W., & Norman, W. H. (1992). Recovery and major depression: Factors associated with twelve-month outcome. American Journal of Psychiatry, 149(1), 93–99. https://doi.org/10.1176/ajp.149.1.93

Koutsouleris, N., Riecher-Rössler, A., Meisenzahl, E. M., Smieskova, R., Studerus, E., Kambeitz, J., ... & Borgwardt, S. (2016). Multisite prediction of 4-week and 52-week treatment outcomes in patients with first-episode psychosis: A machine learning approach. Lancet Psychiatry, 3(10), 935–946. https://doi.org/10.1016/S2215-0366(16)30171-7

de Kovel, C. G. F., van ’t Ent, D., Boomsma, D. I., & Francks, C. (2019). No alterations of brain structural asymmetry in major depressive disorder: An ENIGMA consortium analysis. American Journal of Psychiatry, 176(12), 1039–1049. https://doi.org/10.1176/appi.ajp.2019.18091024

Lam, R. W., Michalak, E. E., Swinson, R., Levitt, A. J., Levitan, R. D., Tam, E. M., ... & Milev, R. (2016). Efficacy of bright light treatment, fluoxetine, and the combination in patients with nonseasonal major depressive disorder: A randomized clinical trial. JAMA Psychiatry, 73(1), 56–63. https://doi.org/10.1001/jamapsychiatry.2015.2235

MacQueen, G. M., Yucel, K., Taylor, V. H., Macdonald, K., Joffe, R., & Milev, R. (2008). Posterior hippocampal volumes are associated with remission rates in patients with major depressive disorder. Biological Psychiatry, 64(10), 880–883. https://doi.org/10.1016/j.biopsych.2008.06.017

Maller, J. J., Daskalakis, Z. J., Fitzgerald, P. B., Freitas, C., Loo, C. K., & Mitchell, P. B. (2018). Increased hippocampal tail volume predicts depression status and remission to antidepressant medications in major depression. Molecular Psychiatry, 23(8), 1737–1744. https://doi.org/10.1038/mp.2017.175

Rush, A. J. (2007). Limitations in efficacy of antidepressant monotherapy. Journal of Clinical Psychiatry, 68(Suppl 1), 8–10. https://doi.org/10.4088/JCP.0901e20c

Rush, A. J. (2009). STAR*D: Revising conventional wisdom. CNS Drugs, 23(8), 627–647. https://doi.org/10.2165/00023210-200923080-00001

Schmaal, L., Hibar, D. P., Sämann, P. G., Hall, G. B., Baune, B. T., Jahanshad, N., ... & Veltman, D. J. (2016). Cortical abnormalities in adults and adolescents with major depression based on brain scans from 20 cohorts worldwide in the ENIGMA Major Depressive Disorder Working Group. Molecular Psychiatry, 22(6), 900–909. https://doi.org/10.1038/mp.2016.60

Schmaal, L., Veltman, D. J., van Erp, T. G., Sämann, P. G., Frodl, T., Jahanshad, N., ... & Hibar, D. P. (2016). Subcortical brain alterations in major depressive disorder: Findings from the ENIGMA Major Depressive Disorder Working Group. Molecular Psychiatry, 21(6), 806–812. https://doi.org/10.1038/mp.2015.69

Schrantee, A., Ruhe, H. G., & Reneman, L. (2020). Psychoradiological biomarkers for psychopharmaceutical effects. Neuroimaging Clinics of North America, 30(1), 53–63. https://doi.org/10.1016/j.nic.2019.10.004

Squarcina, L., Vita, A., Crescini, A., Bellani, M., Brambilla, P., & Trincavelli, M. (2021). Deep learning for the prediction of treatment response in depression. Journal of Affective Disorders, 281, 618–622. https://doi.org/10.1016/j.jad.2020.12.044

Suh, J. S., Schneider, M. A., Adams, R. A., Prashad, S., Barlow, N., Mills, C., ... & Thompson, P. M. (2020). An investigation of cortical thickness and antidepressant response in major depressive disorder: A CAN-BIND study report. NeuroImage: Clinical, 25, 102178. https://doi.org/10.1016/j.nicl.2020.102178

van Velzen, L. S., Kelly, S., Isaev, D., Aleman, A., Aftanas, L., Bauer, J., ... & Schmaal, L. (2020). White matter disturbances in major depressive disorder: A coordinated analysis across 20 international cohorts in the ENIGMA MDD Working Group. Molecular Psychiatry, 25(7), 1511–1525. https://doi.org/10.1038/s41380-019-0477-2

Vythilingam, M., Vermetten, E., Anderson, G. M., Luckenbaugh, D., Anderson, E. R., Snow, J., ... & Bremner, J. D. (2004). Hippocampal volume, memory, and cortisol status in major depressive disorder: Effects of treatment. Biological Psychiatry, 56(2), 101–112. https://doi.org/10.1016/j.biopsych.2004.03.016

Widge, A. S., Zorowitz, S., Basu, I., Paulk, A. C., Cash, S. S., & Eskandar, E. N. (2019). Electroencephalographic biomarkers for treatment response prediction in major depressive illness: A meta-analysis. American Journal of Psychiatry, 176(1), 44–56. https://doi.org/10.1176/appi.ajp.2018.17121358

**Hyperparameter Optimization**

The secondary analysis is concerned with the explicit analysis of several model hyperparameters such as the data representation, clinical predictors and machine learning model. In addition, we optimize a few key algorithm hyperparameters implicitly using Bayesian hyperparameter optimization implemented in SciKit-Optimize (v.0.10.2). Internal cross-validation was regular 5-fold cross-validation. The number of iterations was limited to 25. The parameters optimizes per model and the corresponding search spaces were as follows:

| **Component** | **Hyperparameter** | **Type** | **Range (Space)/Choices** |
| --- | --- | --- | --- |
| KNNImputer | Number of Neighbours | Integer | 1 to 20 |
|  | Weighting Scheme | Categorical | ['uniform', 'distance'] |
| RegressorWrapper | Number of Estimators | Integer | 10 to 200 |
|  | Learning Rate | Real | 0.01 to 0.3 (log-uniform) |
| SelectFromModel (LinearSVC) | Regularization Strength | Real | 1e-3 to 1e2 (log-uniform) |
| SVC | Regularization Strength | Real | 1e-3 to 1e2 (log-uniform) |
|  | Kernel Type | Categorical | ['linear', 'rbf'] |
|  | Polynomial Degree | Integer | 2 to 5 |
| GradientBoostingClassifier | Number of Boosting Stages | Integer | 50 to 500 |
|  | Learning Rate | Real | 0.001 to 0.3 (log-uniform) |
|  | Maximum Depth | Integer | 3 to 15 |
|  | Subsample Fraction | Real | 0.5 to 1.0 |
|  | Maximum Features | Categorical | ['auto', 'sqrt', 'log2', None] |
| LogisticRegression | Regularization Strength | Real | 1e-4 to 1e2 (log-uniform) |
|  | ElasticNet Mixing Ratio | Real | 0 to 1 |
| RandomForestClassifier | Number of Trees | Integer | 50 to 500 |
|  | Maximum Depth | Integer | 5 to 50 |
|  | Maximum Features | Categorical | ['auto', 'sqrt', 'log2', None] |

**Statistical Testing**

Comparisons of models against chance and paired comparisons of models were performed with permutation testing. In permutation testing, the class labels are randomly shuffled to create a null distribution of the test statistic, simulating the scenario where there is no true association between features and labels. There is no universal convention for the calculation of two-sided p-values in permutation. In this work we follow the conservative approximation suggested by Phipson et al. and Ernst et al., rather than the unbiased estimator as suggested by Efron et al. (Philipson et al., 2010; Ernst et al., 2004; Efron et al., 1993). This approximation includes the observed value of the test as an element of the randomized null distribution. Thus, p-values are defined as (C+1) / (n_permutations+1), where C is the number of permutations whose score is higher than or equal to the true score. Note that when calculating the proportion of the randomized null distribution is equal to or higher than the observed value of the test statistic, the values in the numerator and denominator are both increased by one.

Efron, Bradley, and Robert J. Tibshirani. An Introduction to the Bootstrap. Chapman and Hall/CRC, 1994.

Ernst, Michael D. “Permutation Methods: A Basis for Exact Inference.” Statistical Science, 2004, pp. 676–85.

Phipson, Belinda, and Gordon K. Smyth. “Permutation P-Values Should Never Be Zero: Calculating Exact P-Values When Permutations Are Randomly Drawn.” Statistical Applications in Genetics and Molecular Biology, vol. 9, no. 1, 2010.

**Motivation for Machine Learning Pipeline Configurations**

In this study, we use a combination of machine learning models to classify treatment response in MDD based on neuroimaging data and clinical biomarkers. The models we used needed to leverage this data and be able to integrate them. We chose Support Vector Machine (SVM) for its widespread use and effectiveness with structured data. Gradient Boosting was selected for its ability to handle complex relationships within the data effectively. Additionally, we incorporated a state-of-the-art neural network model, ResNET, known for its exceptional performance in image data analysis, which present a novel approach to the analysis of cortical data. By combining these models, we aim to maximize predictive accuracy and gain insights into the relationship between neuroimaging markers, clinical descriptors, and treatment response in MDD.

We added classifiers to Random Forest Classifiers implemented in SciKit-Learn (v.1.1.2). We compared this model to the models we had preregistered to see if it showed better performance. The results of this analysis are presented in supplementary table S10. The mean balanced classification accuracy of random forest classifiers was not statistically different from chance.

All machine learning models were trained in one of two CV methods implemented in SciKit-Learn. The first method was outcome-stratified *K*-fold (SKF) CV, for ten folds. The second method systematically excluded a single cohort from the training set to be used as a test set to assess the generalizability and robustness of our method across cohorts (Leave Site Out Cross Validation; LSO-CV). The use of both SKF-CV and LSO-CV allows us to separate inherent variance from inter-site variance. Specifically, if models perform well in SKF-CV but not in LSO-CV, this suggests that model performance is affected by large inter-site differences. Conversely, if models perform poorly in both, it is likely due to inherent variance in the data. By comparing the results from these two CV approaches, we can gain meaningful insights into the model's generalizability to new sites.

**Exploratory Analysis of Inclusion of Subcortical Data**

To assess the effect of including subcortical data in the prediction models, we repeated our analyses as described in the main manuscript, with some deviations due to the limitation of subcortical data to volumetric measures, thereby preventing a thorough assessment of more sophisticated machine learning models. We therefore tested one data representation, the inclusion of clinical predictors in the model, two machine learning models, and two CV-schemes.

Data representations

Subcortical volumes generated by FreeSurfer were available for eight subcortical regions. These regions are the nucleus accumbens, amygdala, caudate nucleus, hippocampus, globus pallidus, putamen and thalamus. Volumes were available for both hemispheres. In addition, total intracerebral volume was available which was used to normalize subcortical region volumes. This makes for a total 25 additional predictors for these exploratory analyses.

Clinical predictors

Clinical variables included as additional predictors were the same for our exploratory analyses of model performance.

###

Machine learning model

To test different machine learning variations, we trained two types of models: I. Support Vector Classifier (SVC); II. Gradient Boosting Classifier (GBC) implemented in SciKit-Learn. Training the ResNet was not possible, as no spatial representation of the cortical thickness and surface area was available.

Cross-validation methods

All machine learning models were trained in one of two CV methods implemented in SciKit-Learn. The first method was SKF-CV, for ten folds. The second method systematically excluded a single cohort from the training set to be used as a test set to assess the generalizability and robustness of our method across cohorts (LSO-CV).

Primary and secondary analyses

For our primary analysis, we tested if the mean performance of the models was statistically better than chance, as described in the main manuscript.

# Table S1. Performance for Long Treated Patients Compared to the Whole Population.

For this sensitivity analysis, we illustrated the balanced accuracy for each of the machine learning pipeline configurations we investigated.

|  | **Whole population (n=262)** | | **Long Treated (n=119)** | |
| --- | --- | --- | --- | --- |
|  | **Balanced Accuracy** | | **Balanced Accuracy** | |
| **RQ1: Overall performance** | **Mean** | **SD** | **Mean** | **SD** |
| Full population | 50.5 | 5.9 | 50.5 | 5.2 |
|  |  |  |  |  |
| **RQ2-I: Cortical data representations** |  |  |  |  |
| a. ROI average | 50.6 | 5.4 | 49.6 | 4.7 |
| b. Cortical vector | 50.9 | 7.7 | 51.4 | 6.5 |
| c. Cortical thickness projection | 50.3 | 5.0 | 49.4 | 3.6 |
| d. Surface area projection | 49.8 | 2.1 | 51.6 | 3.9 |
|  |  |  |  |  |
| **RQ2-II: Adding clinical data** |  |  |  |  |
| Cortical data only | 51.0 | 4.3 | 50.5 | 5.2 |
| Clinical data added | 50.5 | 5.9 | 52.5 | 7.3 |
|  |  |  |  |  |
| **RQ2-III: Machine learning model** |  |  |  |  |
| Support vector classifier | 50.5 | 4.3 | 50.2 | 1.1 |
| Gradient boosting classifier | 51.0 | 8.3 | 50.8 | 8.1 |
| ResNet | 50.0 | 3.9 | 50.5 | 3.9 |
|  |  |  |  |  |
| **RQ2-IV: Cross-validation method** |  |  |  |  |
| 10-Fold cross-validation | 50.5 | 5.9 | 50.5 | 5.2 |
| Leave-site-out cross-validation | 52.3 | 5.5 | 52.7 | 4.6 |
|  |  |  |  |  |
| **Exploratory I: Subgroup performance** |  |  |  |  |
| Single cohort | 49.6 | 17.1 | 49.6 | 7.1 |
| Response rate selected cohorts | 50.1 | 7.2 | 50.1 | 7.2 |
| Extreme (non-)responders | 63.9 | 10.6 | 63.9 | 10.6 |

**Abbreviations**: ROI: Region of Interest, RQ: Research Question, ResNet: deep learning residual network, SD: standard deviation.

# Table S2. Sensitivity Analysis for Period between Baseline MRI and Treatment Initiation

|  | **Balanced Accuracy** | | **Accuracy** | | **Chance** | | **Different from chance** |
| --- | --- | --- | --- | --- | --- | --- | --- |
|  | **Mean** | **SD** | **Mean** | **SD** | **Mean** | **SD** | **p-value** |
|  |  |  |  |  |  |  | **0.917** |
| Week two cutoff (n=262) | 50.5 | 5.9 | 53.6 | 7.2 | 53.2 | 6.8 | 0.657 |
| Week one cutoff (n=222) | 50.5 | 5.7 | 53.8 | 7.2 | 53.2 | 6.8 | 0.657 |

# Table S3. Information on the MRI Scanners and Acquisition Protocols Used for Each Cohort.

|  | **AFDDIS** | **DEP- ARREST- CLIN** | **Hiroshima** | **Melbourne** | **Minnesota** | **Milano OSR** |
| --- | --- | --- | --- | --- | --- | --- |
| Scanner | Siemens Magnetom TrioTrim | Philips Achieva | Siemens Magnetom Spectra/  GE Signa HDxt/  Siemens Magnetom Verio.Dot | GE Signa Excite | Siemens Tim Trio | Philips Ingenia/ Philips Intera |
| Field strength | 3T | 3T | 3T | 3T | 3T | 3T |
|  |  |  |  |  |  |  |
|  |  |  |  |  |  |  |
| Sequence | 3D T1 | 3D T1 | Siemens: ADNI MPRAGE/ GE: FSPGR | 3D BRAVO | MP-GRE | 3D-MPRAGE |
| Number of slices | 176 | 180 | Siemens: 192/ GE: 184 |  | 224 | 220 |
| TR/TE/TI (ms) | 2250/3.26 | 7000/3500 | 6.8/1.9 | 7900/3000 | 2530/3.65/ 1100 | 2500/4.6 |
| Flip angle |  | 8 | 20 | 13 | 7 |  |
| FOV | 256 | 352x352x180 | 256 | 256 | 256x176 | 230 |
| Voxel size (mm) | 1x1x1 | 0.8x0.8x1.0 | 1x1x1 | 1.0 | 1x1x1 | 0.9x0.9x0.8 |
|  |  |  |  |  |  |  |

**Abbreviations**: MPRAGE: magnetization-prepared rapid acquisition gradient echo; FSPGR: fast spoiled gradient-echo; TR: repetition time; TE: echo time, TI: inversion time.

# Table S4. Pipeline Configuration Variations and Defaults

| **Pipeline Step** | **Variations** | **Default** |
| --- | --- | --- |
| (sub)Population | All patients OR  Response rate selected cohorts OR  Single cohort OR  Extreme (non-)responders | All patients |
| Data representation | ROI average OR  Cortical vector OR  Cortical thickness projection OR  Surface area projection | Mean result |
| Inclusion of clinical data | With clinical data OR  Without clinical data | Without clinical data |
| Inclusion of subcortical data | With subcortical data OR  Without subcortical data | Without subcortical data |
| Imputation | - | 5-nearest neighbors imputer |
| Regressor | - | linear regression of age and age^2^ |
| Harmonization | - | ComBat |
| Standardization | - | StandardScaler |
| feature selector | - | LASSO feature selection |
| classifier | Support Vector Classifier OR  Gradient Boosting Classifier OR  ResNet Classifier | mean result |
| outcome label | - | is_responder |
| cross-validation scheme | 10-fold cross validation OR  Leave-site-out cross-validation | 10-fold cross-validation |

# Table S5. Patient Characteristics of the Extreme (Non-)responders Subpopulation.

Characteristics are shown for the total subpopulation, and for the non-responders and responders used in our exploratory analyses separately. In the first part of the table, the mean and SD per characteristic are provided; in the second part of the table, the participant numbers and the percentage per characteristic are provided.

| **Characteristic** | **Extremes Total (n=132)** | | **Extreme Non-responders**  **(n=66)** | | **Extreme Responders**  **(n=66)** | |
|  | Mean | SD | Mean | SD | Mean | SD |
| **Age (years)** | 34.3 | 14.8 | 35.9 | 16.0 | 32.6 | 13.3 |
| **Treatment duration (weeks)** | 8.9 | 3.3 | 7.9 | 3.2 | 9.8 | 3.2 |
| **Normalized pre-treatment symptom severity** | 0.03 | 0.98 | -0.29 | 0.95 | 0.35 | 0.91 |
| **Age at first depressive episode (years)** | 27.2 | 13.8 | 28.3 | 14.8 | 26.2 | 12.7 |
|  | N | % | N | % | N | % |
| **Female** | 81 | 61 | 41 | 62 | 40 | 60.6 |
| **MDD is recurrent** | 83 | 63 | 44 | 67 | 39 | 59.1 |
| **Responds to treatment** | 66 | 50 | 0 | 0 | 66 | 100 |
| **Uses SSRI** | 91 | 69 | 59 | 89 | 32 | 48.5 |
| **Uses SNRI** | 42 | 32 | 8 | 12 | 34 | 51.5 |
| **Uses atypical antidepressant** | 5 | 4 | 2 | 3 | 3 | 4.5 |
| **In the response rate selected subpopulation** | 67 | 51 | 49 | 74 | 18 | 27.3 |
| **In the extreme (non-)responders subpopulation** | 132 | 100 | 66 | 100 | 66 | 100 |

Abbreviations: MDD: major depressive disorder; SD: standard deviation, SSRI: selective serotonin reuptake inhibitor; SNRI: selective serotonin and norepinephrine reuptake inhibitory.

# Table S6. Patient Characteristics by Treatment Outcome.

Characteristics are shown separately for the non-responders and responders in the total population. In the first part of the table, the mean and SD per characteristic are provided; in the second part of the table, the participant numbers and the percentage per characteristic are provided.

| **Characteristic** | **Total (n=262)** | | **Responders (n=149)** | | **Non-responders (n=113)** | |
| --- | --- | --- | --- | --- | --- | --- |
|  | Mean | SD | Mean | SD | Mean | SD |
| **Age (years)** | 36.5 | 15.3 | 35.6 | 15.0 | 37.6 | 15.6 |
| **Treatment duration (weeks)** | 8.3 | 3.2 | 8.7 | 3.3 | 7.8 | 3.1 |
| **Normalized pre-treatment symptom severity** | 0.04 | 0.99 | 0.18 | 0.97 | -0.15 | 0.99 |
| **Age at first depressive episode (years)** | 30.3 | 14.8 | 30.2 | 14.5 | 30.3 | 15 |
|  | N | % | N | % | N | % |
| **Female** | 154 | 59 | 84 | 56 | 70 | 61.9 |
| **MDD is recurrent** | 160 | 61 | 83 | 56 | 77 | 68.1 |
| **Responds to treatment** | 149 | 57 | 149 | 100 | 0 | 0 |
| **Uses SSRI** | 194 | 74 | 94 | 63 | 100 | 88.5 |
| **Uses SNRI** | 68 | 26 | 53 | 36 | 15 | 13.3 |
| **Uses atypical antidepressant** | 16 | 6 | 9 | 6 | 7 | 6.2 |
| **In the response rate selected subpopulation** | 157 | 60 | 73 | 49 | 84 | 74.3 |
| **In the extreme (non-)responders subpopulation** | 132 | 50 | 66 | 44 | 66 | 58.4 |

**Abbreviations**: MDD: major depressive disorder; SD: standard deviation, SSRI: selective serotonin reuptake inhibitor; SNRI: selective serotonin and norepinephrine reuptake inhibitory.

# Table S7. Performance for the Exploratory Analysis of the Extreme (Non-)responders Subpopulation.

The balanced accuracy, accuracy, and priori chance are provided for each of the machine learning pipeline configurations investigated. On the right, the p-value is provided, which illustrates whether a machine learning pipeline configuration outperforms chance. The overarching p-values for each research question expresses the difference among (using permutational multivariate ANOVA) or between permutation test) the configuration variations tried, e.g. for RQ2-II whether the balanced accuracy of “Cortical data only” differs significantly from “Clinical data added”. Significance was inferred when p<0.05.

|  | **Balanced Accuracy** | | **Accuracy** | | **Chance** | | **Different from chance** |
| --- | --- | --- | --- | --- | --- | --- | --- |
| **RQ1: Overall performance** | **Mean** | **SD** | **Mean** | **SD** | **Mean** | **SD** | **p-value** |
| Full population | 63.9 | 10.6 | 63.6 | 8.7 | 52.1 | 10.3 | 0.001 |
|  |  |  |  |  |  |  |  |
| **RQ2-I: Cortical data representations** |  |  |  |  |  |  | **0.0839** |
| a. ROI average | 65.1 | 8.1 | 65.3 | 6.7 | 53.3 | 10.3 | 0.003 |
| b. Cortical vector | 63.6 | 16.3 | 63.9 | 12.2 | 53.8 | 12.6 | 0.002 |
| c. Cortical thickness projection | 63.1 | 2.4 | 60.5 | 5.2 | 48.1 | 5.7 | 0.010 |
| d. Surface area projection | 63.1 | 2.7 | 62.8 | 5.0 | 50.1 | 6.9 | 0.010 |
|  |  |  |  |  |  |  |  |
| **RQ2-II: Adding clinical data** |  |  |  |  |  |  | **0.703** |
| Cortical data only | 65.4 | 13.2 | 65.1 | 9.7 | 53.6 | 11.6 | 0.001 |
| Clinical data added | 63.9 | 10.6 | 63.6 | 8.7 | 52.1 | 10.3 | 0.001 |
|  |  |  |  |  |  |  |  |
| **RQ2-III: Machine learning model** |  |  |  |  |  |  | **0.300** |
| Support vector classifier | 62.8 | 13.8 | 62.8 | 10.7 | 51.8 | 12.4 | 0.003 |
| Gradient boosting classifier | 65.9 | 11.6 | 66.5 | 8.6 | 55.3 | 10.3 | 0.002 |
| ResNet | 63.1 | 2.6 | 61.7 | 5.2 | 49.1 | 6.4 | 0.001 |
|  |  |  |  |  |  |  |  |
| **RQ2-IV: Cross-validation method** |  |  |  |  |  |  | **0.703** |
| 10-Fold cross-validation | 63.9 | 10.6 | 63.6 | 8.7 | 52.1 | 10.3 | 0.001 |
| Leave-site-out cross-validation | 65.0 | 7.6 | 56.7 | 14.5 | 41.0 | 18.2 | 0.001 |

**Abbreviations**: ROI: Region of Interest; RQ: Research Question; ResNet: deep learning residual network, SD: standard deviation.

# Table S8. Feature Contributions in the Subpopulation of Extreme (Non-)responders

Feature importances for the prediction of treatment outcome were normalized to range from 0 to 1 for use as scalars for the prediction models for each of the labels of the Desikan-Killiany Atlas for the left and right hemisphere. The sign indicates the direction of the relationship between a positive treatment response and either cortical thickness or surface area. Red indicates a negative direction, whilst green indicates a positive direction. The magnitude (visualized as saturation) of the coefficients indicates the strength of the relationship.

| **Desikan-Killiany Cortical Label** | **Cortical Thickness** | | **Surface Area** | |
| --- | --- | --- | --- | --- |
|  | **Left** | **Right** | **Left** | **Right** |
| Banks STS | 0.226 | 0.725 | 0.401 | 0.319 |
| Caudal Anterior Cingulate | 0.715 | 0.237 | 0.733 | 0.533 |
| Caudal Middle Frontal | 0.717 | 0.548 | 0.559 | 0.430 |
| Cuneus | 0.554 | 0.594 | 0.519 | 0.218 |
| Frontal Pole | 0.717 | 0.505 | 0.514 | 0.592 |
| Fusiform | 0.694 | 0.174 | 0.114 | 0.370 |
| Inferior Parietal | 0.622 | 0.953 | 0.667 | 0.645 |
| Inferior Temporal | 0.263 | 0.311 | 0.000 | 0.413 |
| Insula | 0.434 | 0.185 | 0.656 | 0.817 |
| Isthmus Cingulate | 0.348 | 0.361 | 0.567 | 0.514 |
| Lateral Occipital | 0.529 | 0.290 | 0.814 | 0.444 |
| Lateral Orbitofrontal | 0.617 | 0.384 | 0.347 | 0.208 |
| Lingual | 0.746 | 0.586 | 0.606 | 0.394 |
| Medial Orbitofrontal | 0.355 | 1.000 | 0.682 | 0.421 |
| Middle Temporal | 0.418 | 0.234 | 0.171 | 0.277 |
| Paracentral | 0.407 | 0.210 | 0.132 | 0.546 |
| Parahippocampal | 0.968 | 0.101 | 0.118 | 0.128 |
| Pars Opercularis | 0.114 | 0.617 | 0.475 | 0.746 |
| Pars Orbitalis | 0.819 | 0.571 | 0.682 | 0.844 |
| Pars Triangularis | 0.172 | 0.701 | 0.408 | 0.449 |
| Pericalcarine | 0.212 | 0.196 | 0.469 | 0.336 |
| Postcentral | 0.617 | 0.469 | 0.655 | 0.575 |
| Posterior Cingulate | 0.643 | 0.297 | 0.641 | 0.465 |
| Precentral | -0.550 | -0.550 | 0.849 | 1.000 |
| Precuneus | 0.765 | 0.892 | 0.487 | 0.586 |
| Rostral Anterior Cingulate | 0.765 | 0.502 | 0.772 | 0.288 |
| Rostral Middle Frontal | 0.000 | 0.466 | 0.262 | 0.255 |
| Superior Frontal | 0.768 | 0.838 | 0.655 | 0.690 |
| Superior Parietal | 0.544 | 0.780 | 0.385 | 0.256 |
| Superior Temporal | 0.208 | 0.568 | 0.434 | 0.493 |
| Temporal Pole | 0.210 | 0.663 | 0.400 | 0.543 |
| Transverse Temporal | 0.279 | 0.544 | 0.472 | 0.157 |

# Table S9. Model Performance for Exploratory Analyses of the Inclusion of Subcortical Data.

The balanced accuracy. accuracy. and priori chance are provided for each of the machine learning pipeline configurations investigated. On the right. the p-value is provided. which illustrates whether a machine learning pipeline configuration outperforms chance. The overarching p-values for each research question expresses the difference among (using permutational multivariate ANOVA) or between (permutation test) the configuration variations tried. e.g. for RQ2-II whether the balanced accuracy of “Cortical data only” differs significantly from “Clinical data added”. Significance was inferred when p<0.05.

|  | **Balanced Accuracy** | | **Accuracy** | | **Chance** | | **Different from chance** | |
| --- | --- | --- | --- | --- | --- | --- | --- | --- |
|  | **Mean** | **SD** | **Mean** | **SD** | **Mean** | **SD** | **p-value** | |
| **Combinations of cortical ROI. subcortical and clinical data in the whole population** | | | | | | | | **0.306** |
| Only cortical | 50.5 | 5.9 | 53.6 | 7.2 | 53.2 | 6.8 | 0.657 | |
| Only clinical | 53.6 | 4.8 | 55.9 | 4.2 | 54.3 | 6.2 | 0.289 | |
| Only subcortical | 54.1 | 4.4 | 56.0 | 5.3 | 54.5 | 6.2 | 0.217 | |
| Cortical and clinical | 51.0 | 5.5 | 54.4 | 6.3 | 54.2 | 5.7 | 0.617 | |
| Cortical and subcortical | 51.6 | 3.7 | 55.3 | 3.8 | 55.4 | 5.7 | 0.587 | |
| Subcortical and clinical | 53.4 | 5.0 | 55.7 | 5.8 | 55.2 | 5.9 | 0.554 | |
| (Sub)cortical and clinical | 55.6 | 5.0 | 57.6 | 5.4 | 55.9 | 5.6 | 0.275 | |
| **Combinations of cortical ROI. subcortical and clinical data in the Extreme (Non-)responder population** | | | | | | | | **0.017** |
| Only cortical | 63.9 | 10.6 | 63.6 | 8.7 | 52.1 | 10.3 | 0.001 | |
| Only clinical | 67.4 | 8.6 | 67.2 | 6.8 | 52.9 | 10.5 | 0.002 | |
| Only subcortical | 68.3 | 9.6 | 68.0 | 7.3 | 52.8 | 10.3 | 0.001 | |
| Cortical and clinical | 65.4 | 13.2 | 65.1 | 9.7 | 53.6 | 11.6 | 0.001 | |
| Cortical and subcortical | 63.0 | 12.3 | 62.8 | 8.7 | 53.6 | 10.0 | 0.009 | |
| Subcortical and clinical | 69.2 | 9.6 | 69.6 | 7.3 | 53.0 | 9.8 | 0.001 | |
| (Sub)cortical and clinical | 71.0 | 10.2 | 70.9 | 7.6 | 53.0 | 9.7 | 0.001 | |

**Abbreviations:** ROI: Region of Interest; RQ: Research Question. SD: standard deviation.

# Table S10. Exploratory Analysis using Random Forests

The outcome of this analysis can also be found as Exploratory Analysis III in section 6.3.3 of the online code repository (Poirot, Boucherie, Reneman & Schrantee. 2023).

|  | **Balanced Accuracy** | | **Accuracy** | | **Chance** | | **Different from chance** |
| --- | --- | --- | --- | --- | --- | --- | --- |
| **Classifier** | **Mean** | **SD** | **Mean** | **SD** | **Mean** | **SD** | **0.220** |
| Support vector classifier | 50.5 | 4.3 | 55.9 | 4.3 | 55.8 | 3.8 | 0.545 |
| Gradient boosting classifier | 51.0 | 8.3 | 53.8 | 7.4 | 53.7 | 6.5 | 0.701 |
| ResNet | 50.0 | 3.9 | 51.0 | 8.3 | 50.1 | 8.2 | 0.372 |
| Logistic regression classifier | 51.5 | 6.3 | 52.7 | 6.2 | 49.9 | 6.1 | 0.199 |
| Random forest classifier | 52.1 | 4.8 | 55.3 | 5.2 | 54.3 | 6.4 | 0.463 |
